# Ultrafast Dynamics Revealed with Time-Resolved Scanning Tunneling Microscopy: A Review

**DOI:** 10.1021/acsaom.2c00169

**Published:** 2023-03-17

**Authors:** Kangkai Liang, Liya Bi, Qingyi Zhu, Hao Zhou, Shaowei Li

**Affiliations:** † Department of Chemistry and Biochemistry, University of California, San Diego, La Jolla, California 92093-0309, United States; ‡ Materials Science and Engineering Program, University of California, San Diego, La Jolla, California 92093-0418, United States

**Keywords:** scanning tunneling microscopy, pump−probe spectroscopy, nanoscale ultrafast dynamics

## Abstract

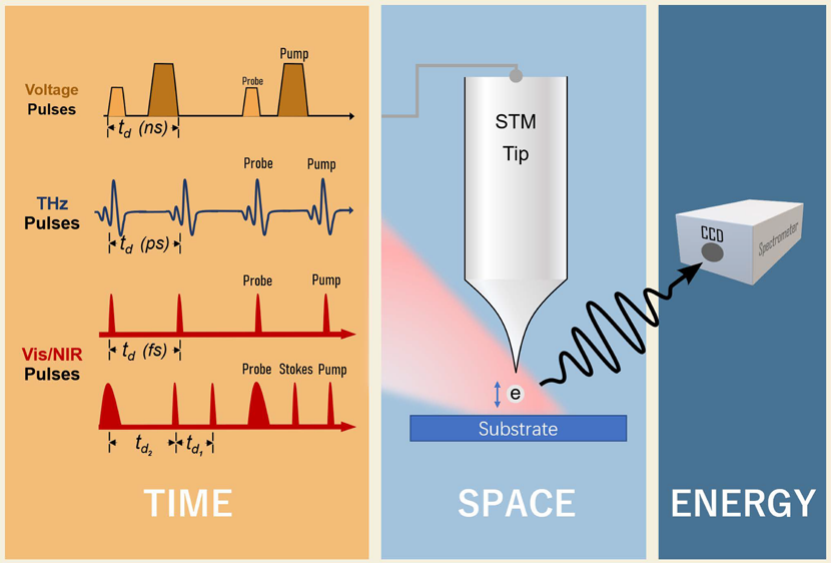

A scanning tunneling microscope (STM) capable of performing pump–probe spectroscopy integrates unmatched atomic-scale resolution with high temporal resolution. In recent years, the union of electronic, terahertz, or visible/near-infrared pulses with STM has contributed to our understanding of the atomic-scale processes that happen between milliseconds and attoseconds. This time-resolved STM (TR-STM) technique is evolving into an unparalleled approach for exploring the ultrafast nuclear, electronic, or spin dynamics of molecules, low-dimensional structures, and material surfaces. Here, we review the recent advancements in TR-STM; survey its application in measuring the dynamics of three distinct systems, nucleus, electron, and spin; and report the studies on these transient processes in a series of materials. Besides the discussion on state-of-the-art techniques, we also highlight several emerging research topics about the ultrafast processes in nanoscale objects where we anticipate that the TR-STM can help broaden our knowledge.

## 1 Introduction

The invention of scanning tunneling microscopy (STM) has allowed for unprecedented spatial resolution down to the atomic scale because of the exponential relationship between quantum tunneling probability and the distance between the tip and a substrate.^1^ It was soon developed into a versatile tool that provides molecular-level insights into various physical chemistry problems, including chemical structure,^2−15^ electronic orbital,^16−20^ vibration,^21−29^ atomic manipulation,^30−37^ potential energy surface characterization,^38^ and spin detection.^39−56^ Additional functions with enhanced time resolution, such as video-rate faster scanning^57−59^ and spatial atom-tracking,^60^ were later developed for direct visualization of the dynamical motions of adsorbates.^61−64^ However, in most cases, the time resolution of STM is limited to the microsecond scale by the response time of its feedback electronics and, thus, can hardly contribute to our understanding of the chemical dynamics that often happen at the time scale of picosecond or femtosecond. There has been a desire to turn STM into a fast-responding camcorder with subpicosecond sensitivity to capture ultrafast processes with atomic-scale details.

The development of the pump–probe technique with either laser or electron pulses has made it possible to induce dynamic transitions and follow their temporal evolution. It has contributed to a greater understanding of the intermediate processes of various quantum degrees of freedom, such as vibrations,^65−70^ electronic orbitals,^71−77^ and electron or nuclear spins in various materials.^78−82^ Moreover, in optical pump–probe measurements, it is possible to manipulate the interference between different excited states by adjusting the phase, frequency, or intensity of the driving laser pulses and consequently control the reaction’s pathway coherently to maximize the yield of the desired product.^83^ It becomes especially powerful in tracking the movement of electrons between molecular states to understand and control chemical transitions. However, because of the diffraction-limited spatial resolution,^84^ most of these studies can only assess the homogeneous properties of an ensemble and cannot account for the nanoscale variation of the local chemical environment.

The exotic chemical and physical phenomena that occur at the nanometer scale have boosted the development of nanoscience and technology in the past two decades. As the size of a material is reduced, the quantum mechanical principles dominate the material behaviors and lead to distinct properties compared with the bulk counterparts. Characterization of the dynamic processes within the local chemical environment is therefore especially essential in the studies of nanoscale physics or chemistry, such as quantum dots and single-atom catalysis. The combination of STM and pump–probe measurement enables the visualization of ultrafast dynamics at the single-atom or single-molecule level. It provides a unique platform where the inhomogeneous activities of individual molecules or low-dimensional materials can be tracked in both space and time, which otherwise are embedded in the ensemble average.^85,86^ In this review, we investigate the approaches that integrate the pump–probe scheme with STM and discuss the recent studies revealing key insights into the dynamics of three types of quantum states: vibration,^87−92^ orbital,^93−97^ and spin.^43,98,99^

## 2 Three Schemes of Time-Resolved Scanning Tunneling Microscopy

### 2.1 All-Electronic Pump–Probe STM

**Figure 1:**
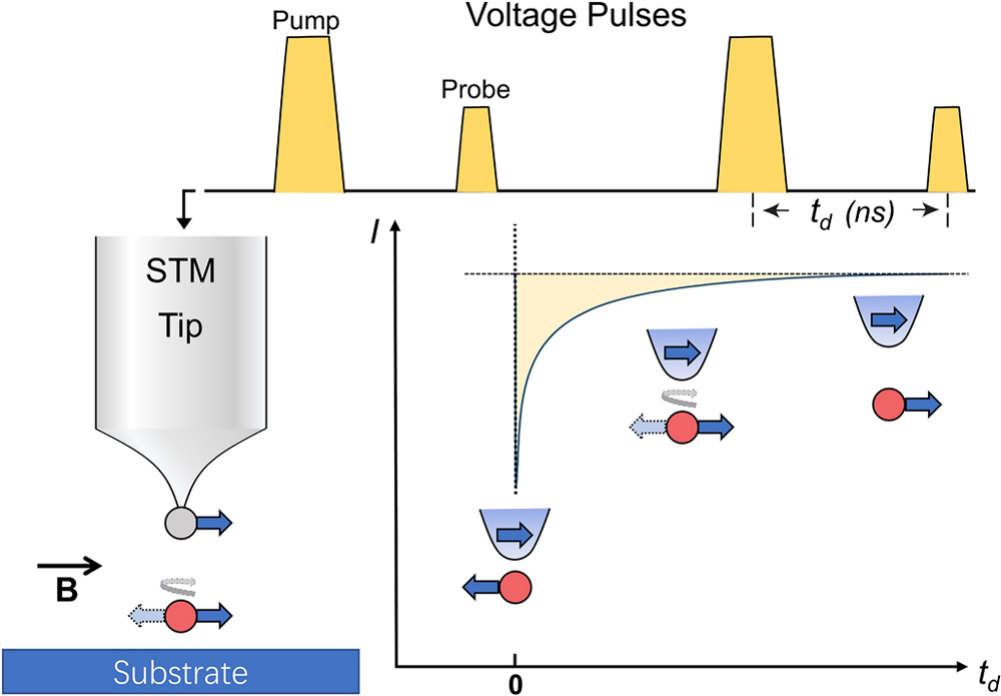
Schematic of the all-electronic pump–probe STM to detect the spin relaxation dynamics of a surface absorbate. The first voltage pulse alternates the spin orientation of the atom or molecule in the STM junction. The spin relaxation dynamics of the excited adsorbate is sensed by spin-polarized current induced by the second pulse.

Traditionally, STM in the constant current mode scans the sample surfaces with the feedback circuit to keep the tunneling current at a constant set point. This is similar to a laser operating in continuous wave mode. However, to achieve higher temporal resolution, the electronic pump–probe scheme can be applied, which uses two short pulses of electrons (Figure 1). The first electron pulse, usually with relatively higher energy, excites the surface adsorbate under the tip into a transition state. The second pulse arrives after an adjustable time delay and interacts with the excited adsorbate. The delay-dependent tunneling current induced by the second pulse contains information directly related to the time evolution of the transition state. In 2010, Loth et al. first combined STM with an electron pulse generator to realize this all-electronic pump–probe scheme^98^ and applied it to probe the spin relaxation of a series of magnetic adsorbates in an external magnetic field. The spin orientation is detected with a spin-polarized tip, which is often made by attaching a magnetic atom, such as Mn, to a nonmagnetic STM tip. The spin orientation of the magnetic atom on the tip aligns nearly parallel with the external magnetic field. Because of the Pauli exclusion principle, preferable tunneling occurs when the orientation of the spin on the tip aligns with the one on the surface. Therefore, when the surface spin relaxes from an excited state triggered by the pump pulse, the time evolution can be traced with the spin-polarized current generated by the probe pulse. This all-electronic pump–probe approach has readily achieved a time resolution in the nanosecond to millisecond range and made great impacts in studying the electron and nuclear spin evolution of material surfaces and absorbates.^70,100−102^

### 2.2 Terahertz Pump–Probe STM

**Figure 2:**
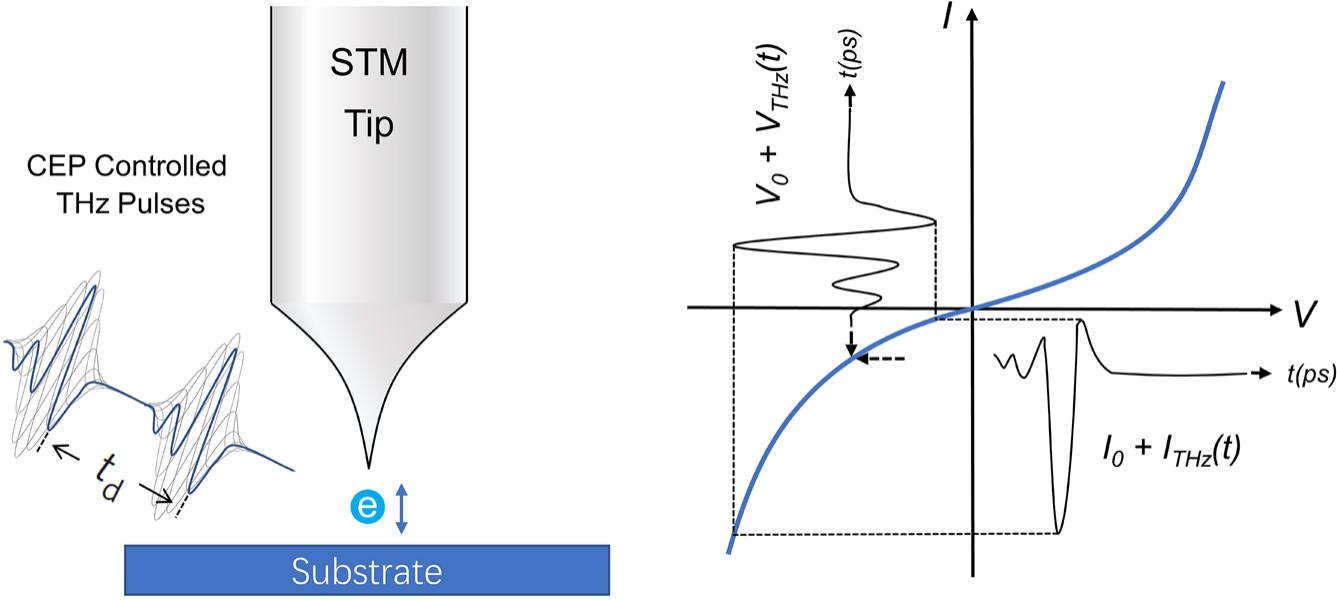
Schematic of the THz-induced electron tunneling at the STM junction. A THz pulse applies a transient voltage at the STM junction to induce ultrafast electron tunneling. This voltage variance from a strong electric field leads to a nonlinear time-dependent tunneling current *I*_THz_(*t*), which allows STM to measure the time-average shift in the tunnel current.

The terahertz (THz) techniques support a new development area of time-resolved STM (TR-STM). Free space THz pulses can be generated as short as a single optical cycle, thereby giving a subpicosecond time resolution. Suboptical cycle resolution can be achieved with two identical THz pulses in autocorrelation.^89,93^ Thanks to the strong electric field and low photon energy, the coupling of THz light pulses with STM avoids the limitation from tip thermal expansion, electronic microstrip bandwidth, and electrostatic coupling.^93,103,104^ Another advantage of THz STM is that it can provide a stable carrier–envelope phase (CEP) in a relatively cheaper and easy-access approach. This is essential for studying coherent interference between light and quantum states.^96^ The working principle of THz STM is detailed in Figure 2. When THz light is focused on the tip apex, the STM junction acts like an antenna that enhances the evanescent THz field, which modulates the Fermi level alignment between sample and tip as ultrafast voltage transients. The enhanced THz field can generate tunnel electrons to either excite the sample underneath the tip or probe its time evolution.^93^ Electron tunneling is commonly involved in both the pump and probe processes, which guarantee a sub-Angstrom-level spatial resolution.^105^ The CEP stability can also help reach a time resolution below the duration of one THz pulse. For example, Cocker et al. demonstrated an access-state-selective tunneling mechanism where the light-induced tunneling only occurs at the peak of the THz pulse, thereby allowing for a temporal resolution shorter than one oscillation period of a THz wave.^89^ However, because of the natural constraint from the optical period, the achievement of a time resolution below a few hundred femtoseconds is still not straightforward.^93^

### 2.3 Visible or Near-Infrared Pump–Probe STM

**Figure 3:**
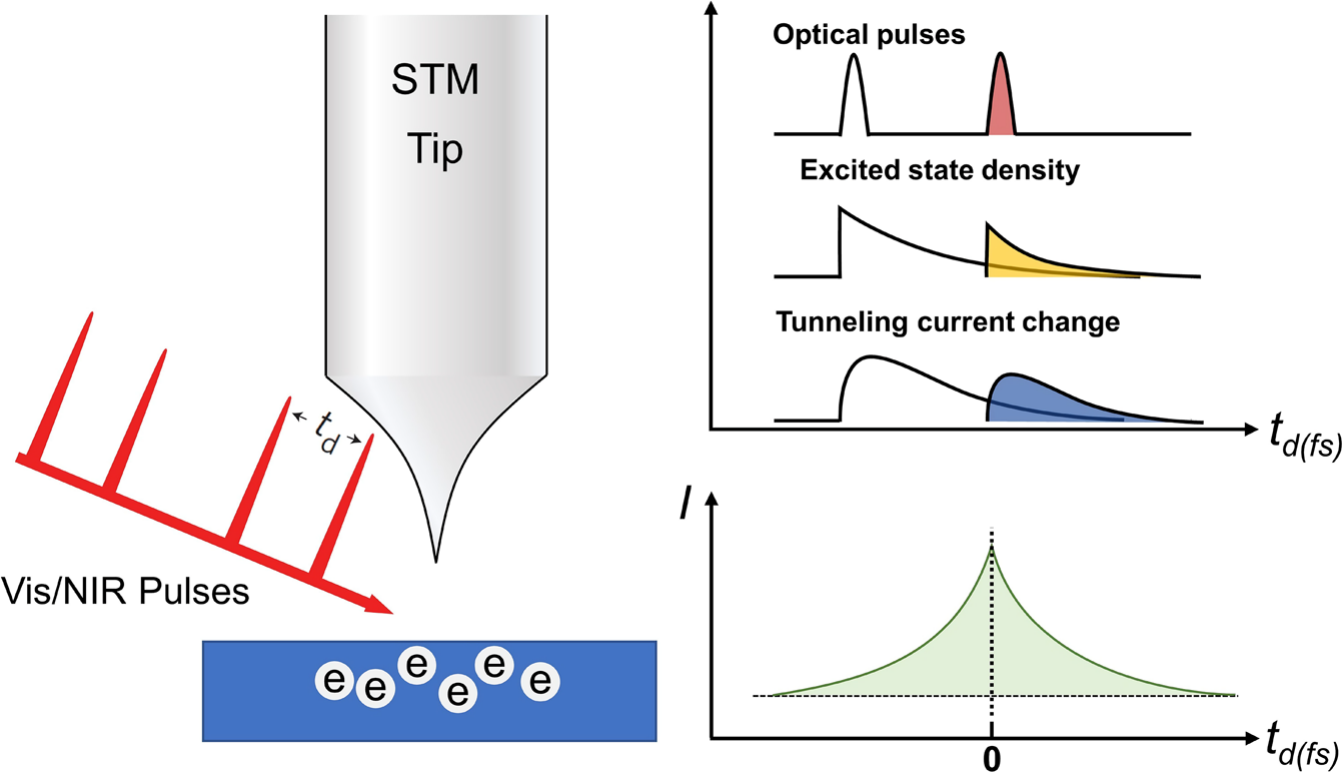
Schematic of the vis/NIR pump–probe STM. The first optical pulse excites the sample into an excited state whose density decays over time. STM reads this density through tunneling current change. The tunnel current induced by the second optical pulse depends on the delay time and reveals the dynamical information on the excited state.

While the time resolution of THz STM is jammed near ∼0.2 ps, which is nearly 2 orders of magnitude longer than the achievable pulse duration in the state-of-art femtosecond laser technology,^89,106^ another laser STM setup can make up for this regret. The visible/near-infrared (vis/NIR) ultrafast laser can readily provide extremely short femtosecond light pulses and has recently been applied in the pump–probe measurement with STM. In most cases, the sample interacts with the electron or THz pulses by coupling with the local electric field at the STM junction, while the vis or IR light can easily excite the sample through photon absorption. For example, in the setup used by Terada et al., pulse trains were generated by two synchronized Ti/Sapphire lasers to illuminate the sample beneath the STM tip with a tunable time delay.^107^ The pump–probe measurement tracks the time correlation between the transient state initiated by the pump pulse and the variation in a local STM measurable induced by the probe pulses (Figure 3). The pump pulses can excite the entire area illuminated by the beam, but the probe processes rely on the detection of photoexcited electron tunneling and, therefore, break the diffraction limit. Nevertheless, short IR pulses can also couple the sample through the field-driven mechanism. Garg et al. advanced the time resolution into a few femtoseconds by focusing a CEP of two-cycle long (<6 fs) optical pulses on the tunneling junction via off-axis parabolic mirrors and capturing the generation of excited electrons on the basis of the interference process of the two pulses.^96^ Recently, femtosecond scale resolution has also been reported in the mid-infrared region through a light-wave-driven mechanism.^108^ So far, the vis/NIR or Mid-Infrared coupled STM has provided the highest time resolution in TR-STM studies. The challenge of this approach often comes from the thermal fluctuations of the STM junction. Unlike the THz case where most materials have a high reflection, the absorption of vis/NIR by either tip or sample can cause thermal expansion, hence interfering with the tunneling measurement. This has posed a great challenge for pump–probe studies where power modulation often needs to be applied on the probe pulse for lock-in measurement. Increasing the laser repetition rate to GHz can partially solve this issue since it reduces the energy of single pulses while still generating a detectable signal.^88^ Other approaches, such as modulating the duration,^109^ frequency,^109,110^ delay time,^107^ or polarization^109^ of the pulses, are applied to avoid change to the laser power. Alternatively, the detection of a signal that is less sensitive to thermal fluctuations, such as molecular motion/reaction and photoexcited current, can also loosen the requirement of thermal stability.^88,106^ Besides, the use of vis/NIR light as a pump and THz light as a probe could potentially unify the advantages of both techniques.^93,95^

Another newly developed approach to detect ultrafast dynamics at the nanoscale is the time-resolved tip-enhanced Raman spectroscopy (TR-TERS) with ultrashort vis/NIR laser pulses.^111−113^ Instead of tunneling electrons detection, TERS collects the scattered photons localized by the filed enhancement at the STM junction.^25,113−115^ Plasmonic materials, such as Ag and Au, are often used for the tip to promote strong light–matter interaction at the STM junction. A submolecular spatial resolution has been reported in STM TERS measurement because of the strongly localized nature of surface plasmons. Meanwhile, the high-energy resolution of TERS makes it ideal to investigate low-energy vibrations. Over the past decade, many studies, such as the vibrational mapping,^25^ structural and chemical changes of single molecules,^28,115,116^ and energy transfer between molecules, have been done with CW light-coupled TERS.^26^ Recently, the combination of ultrafast pump–probe spectroscopy with STM-TERS measurement has provided a new route to capture vibrational dynamics with joint spatial–temporal resolution.^92^

## 3 Recent STM Studies on Ultrafast Dynamics

### 3.1 Nucleus Dynamics

The chemical processes often involve the dynamical rearrangement of atoms in materials, which occurs through different mechanisms, such as vibration,^117,118^ electronic excitation,^96,110,119^ and proton tunneling.^120,121^ Molecular vibrations refer to the periodic motions of atoms relative to one another within a molecule, such that the center position of the mass of the molecule is unchanged. The collective nucleus motion in a periodic lattice can travel across a large-scale sample, which is also known as phonon propagation. The absorption of energy by a vibrator in its ground state can trigger the transitions to a higher vibrational level, a process that can be described with a semiclassical harmonic oscillator model. The vibrational populations can either be read by STM observables, such as tunneling electrons and molecular transition rate, or scattered photons through Raman scattering. The lifetime of molecular vibration is typically at the femtosecond to picosecond level because of the fast energy transfer to the environment—the so-called “bath” coupling.^122^ This time scale exceeds the inherent limitations posted by the electronic bandwidth in traditional STM measurements. In a less common case, nuclei motion can also be due to quantum tunneling.^123,124^ For small atoms like hydrogen, the spatial tunneling of atoms between different energy wells results in the rearrangement of chemical structure.^125^ In this section, we will survey the recent applications of TR-STM on the nucleus dynamics related to vibration, phonon, or quantum tunneling.

In 2016, Cocker et al. for the first time detected the femtosecond-scale single electron tunneling from a surface-adsorbed molecule and recorded the vertical molecular vibration excited by the THz pulses.^89^ In this experiment, ultrafast electron tunneling from the highest occupied molecular orbital (HOMO) of the pentacene to NaCl/Au(110) was triggered by the enhanced THz electric field at the STM junction. It was revealed via the THz pump–probe measurement that the tunneling current induced by the probe pulses oscillates at a frequency of ∼0.5 THz, which was explained as the result of vertical molecular vibration due to temporary ionization by the pump pulses.

Vis/NIR pump–probe STM can also be used to extract the ultrafast nucleus motion of a single molecule. In 2017, Li et al. reported the reversible conformational transition of the pyrrolidine molecules adsorbed on a Cu(001) surface combined with Angstrom–femtosecond resolution.^88^ They found that the transition rate of the molecule under laser illumination shows decaying oscillatory behavior by varying the delay time between the two optical pulses (800 nm, 35 fs pulse width). The peaks in the frequency spectrum at ∼6.9 and ∼2.7 THz were attributed to the bending (27.2 meV) and bouncing (11.3 meV) motions of the pyrrolidine, respectively. It was also demonstrated that with another molecule in proximity, the ∼6.9 THz mode downshifts to ∼6 THz, thereby showcasing the influence of intermolecular interaction on the nucleus dynamics.

In 2020, Peller et al. accomplished the detection and manipulation of ultrafast in-plane vibration-mediated molecular rotation with a THz STM pump–probe scheme.^87^ In the experiment, the bistable magnesium phthalocyanine (MgPc) on NaCl/Cu(111) acted as the single-molecule switch that could be triggered by electron tunneling into the lowest unoccupied molecular orbital (LUMO) of MgPc. In the pump–probe measurement, the MgPc was first perturbed by a weak pump THz pulse and then charged by the strong probe THz- pulse-induced tunneling electron from the tip. The switching probability unambiguously oscillates at ∼0.3 THz when varying the pump–probe delay time, rationalized as the mediation through in-plane rotation excited by the pump pulse.

**Figure 4:**
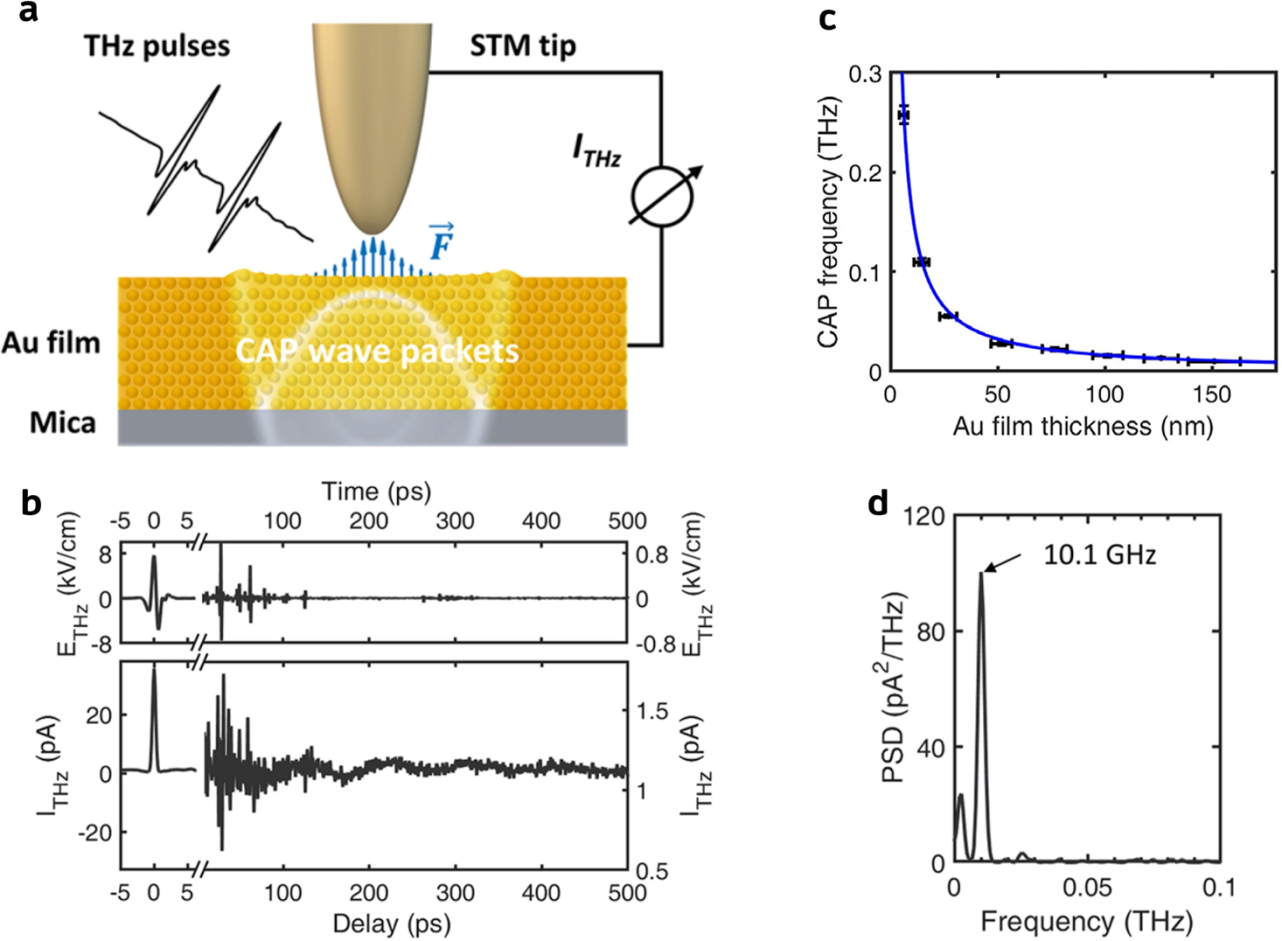
Coherent acoustic phonon (CAP) wave packets in Au thin films. (a) Schematic of the THz pulses–launched CAP wave packets in the Au thin film on mica. (b) Transient THz electric field waveform *E*_THz_ (Top) and probe THz induced tunneling current *I*_THz_ (bottom) collected over a 151 ± 12 nm thick Au film. (c) The CAP frequency *f*_CAP_ fits the function *f*_CAP_ = *v*/2*d* with Au thickness *d* and the speed of longitudinal acoustic phonon *v* in Au. (d) Fast Fourier transform of the time trace of *I*_THz_ in (b). Reproduced with permission from ref (91). Copyright 2022 American Physical Society.

A recent report by Sheng et al. highlights the application of THz pump–probe STM in exploring and controlling the coherent acoustic phonon (CAP),^91^ which is the collective motions of atoms in a lattice. In the experiment, they performed pump–probe measurement on the Au thin film grown on mica with paired, delayed, but identical THz pulses (Figure 4a). The tunneling current induced by the tip-enhanced probe THz electric field shows long-period oscillation at ∼10.1 GHz and decays after several hundreds of picoseconds, which strongly suggests the periodic out-of-plane motion of the Au surface atoms (Figure 4b,d). The observed oscillation was assigned to the CAP wave packets propagating between the Au/mica and Au/vacuum interfaces, whose dispersion relation in a lattice with a thickness of d, *f*_CAP_ = *v*/2*d*, fits well with the oscillation frequencies measured over an Au film of different thicknesses, from 151 nm down to 6.4 nm (Figure 4c). The CAP was rationalized as a consequence of the Au atoms’ displacement by the local ultrafast Coulomb force between the charges on the tip and Au surfaces, which were induced by the strong THz field at the tunnel junction (Figure 4a). It was further shown that the displacement of the surface atoms increased from 2.8 to 5.1 pm when the tip-Au distance decreased by 2.7 Å, which demonstrated a way to manipulate the CAP wave packets. More recently, the coherent phonon modes in another system, ZnO thin films on Ag(111), were revealed with a NIR pump–probe STM.^106^

The CAP can also be excited in graphene nanoribbons (GNRs) through stimulated Raman scattering. Luo et al. demonstrated the observation and control over the CAP in single GNR on Au(111) with a TR-TERS setup.^92^ In this experiment, the GNR under a Au tip was pumped to a superposition of different vibrational states via tip-enhanced impulsively stimulated Raman scattering (ISRS) with two ∼100 fs broadband laser pulses that centered at 780 nm (pump pulse) and 850 nm (Stokes pulse), respectively. The frequency difference between the pump and Stokes pulses (∼2464 cm^–1^) covered several phonon modes to trigger the coherence phonon. A narrowband (∼500 fs and centering at 728 nm) pulse was sequentially used as a probe to interact with the phonon wave packet. The anti-Stokes Raman scattered light of the probe pulse contained the dynamic information on the CAP. By keeping the pump and Stokes pulses temporally overlapped and by varying the delay time (τ_23_) between the Stokes and probe pulses, the dephasing time of the impulsively excited phonons was found to be ∼440 fs. Furthermore, the initial phase of the coherent photon could be manipulated by temporally separating the pump and Stokes pulses (τ_12_) and detecting immediately (τ_23_ = 0) with the probe pulse. The scattered light showed an oscillatory pattern as a function of τ_12_. Fourier transforming at different energies further revealed several beat frequencies, which were assigned to the quantum beating between different vibrational levels.

Besides the dynamic motion of atoms associated with vibration or phonon, the small atoms in a molecule can also rearrange through quantum tunneling. In a recent work by Wang et al., ultrafast THz-coupled STM was adopted to detect the transition between two quantum levels of H_2_ in the STM junction.^90^ The H_2_ could be confined in an asymmetric double-well potential defined by the tip and CuN/Cu(100) substrate. The energy profile of the double-well, as well as the proton tunneling rate across the barrier, codefined two nondegenerate quantum levels in the two spatially separated wells. The transition between these two levels, accompanied by the structural change of H_2_ by hydrogen tunneling, occurred when the molecule was excited with either photons or electrons. In a pump–probe scheme, the authors drove the transition between these two levels with THz pump pulses and extracted its evolving superposition state with delayed probe pulses. The rectification current by the probe pulses, which they showed to be proportional to the second derivative of tunneling current over bias voltage (*d*^2^*I/dV*^2^), exhibited obvious oscillating features, which were attributed to the coherent superposition of the two wave functions.

### 3.2 Electron Dynamics

The excitation and relaxation of electrons between molecular orbitals or semiconductor bands is the origin of many energy and mass transfer processes, including chemical reactions,^126,127^ electrical conduction,^94,99^ and photon emission.^96,128^ The atomic details about the electronic dynamics are vital to understanding the effect of the local chemical environment on these dynamics, such as the effects of nanocatalysis in chemical transformation or atomic dopants in a semiconductor.^117^ In the following section, we will review the recent TR-STM studies on electron dynamics in molecules,^95^ semiconductors,^94,97^ and metals.^96^

**Figure 5:**
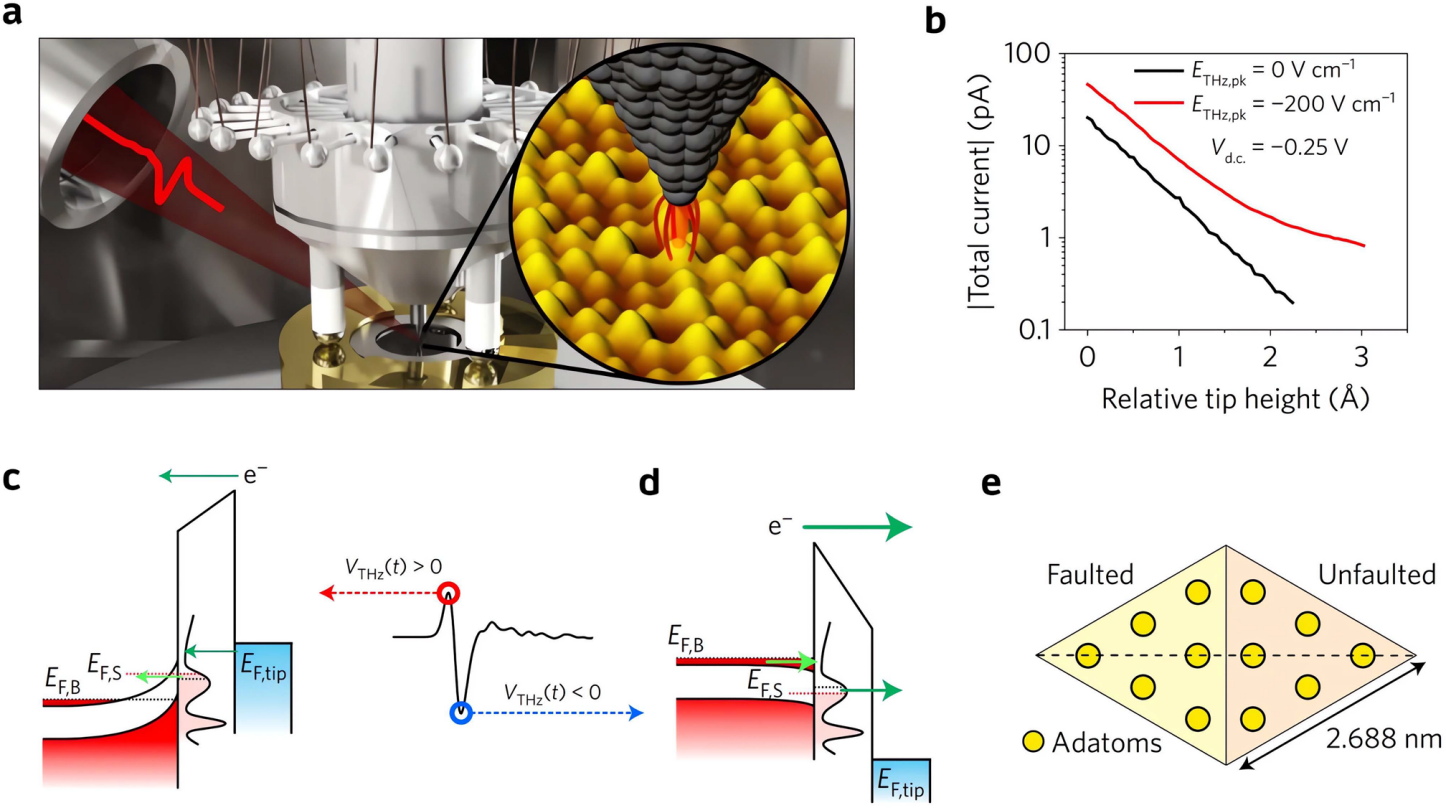
Subpicosecond electron tunneling through adatoms on Si(111)-(7×7) (a) Schematic of the tunneling current induced by THz pulses at the STM tunnel junction. (b) Absolute value of the total tunneling current as a function of relative tip height with (red curve) and without (black curve) the THz illumination at a DC bias of −0.25 V. (c,d) Schematics of ultrafast electron tunneling processes at the (c) positive and (d) negative half-cycles of the THz pulse, respectively. *E*_F,tip_, *E*_F,S_, and *E*_F,B_ are the Fermi levels of the tip, Si(111)-(7×7) surface states, and Si bulk states, separately. (e) Unit cell of the Si(111)-(7×7) reconstruction. The higher local density of states of the faulted half close to the Fermi level make it look higher than the unfaulted half in an STM constant-current image at the negative bias. Reproduced with permission from ref (94). Copyright 2017 Nature Publishing Ltd.

**Figure 6:**
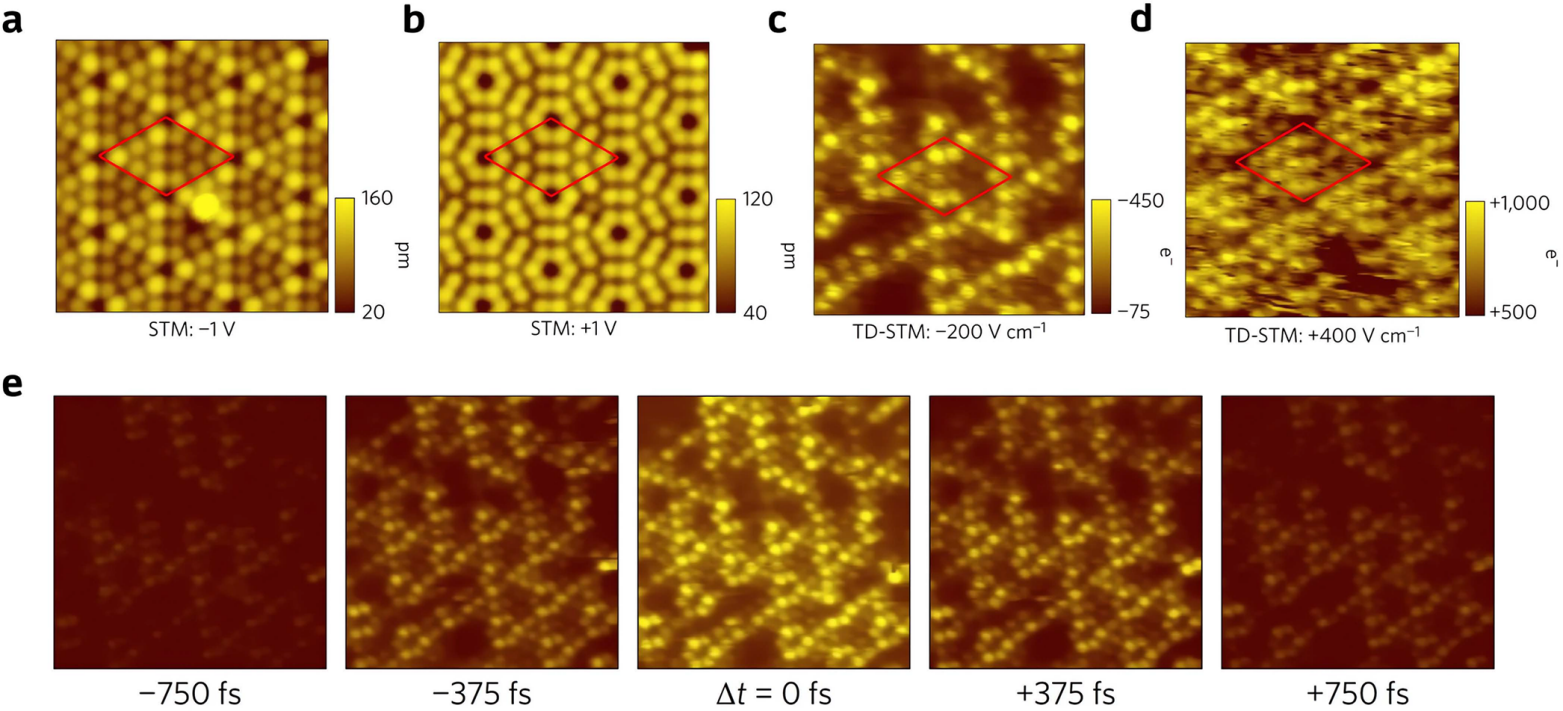
Conventional and THz-driven STM imaging of Si(111)-(7×7). (a,b) STM constant-current images of Si(111)-(7×7) over the same area at −1 and +1 V, respectively. (c,d) Constant-height mapping of THz-induced tunneling current over the same area as in (a,b) with negative and positive THz electric field, respectively. V_d.c._= 0 in both cases. (e) Constant-height mapping of THz autocorrelation signal at different delay times. The contrast in the images is the number of electrons per pulse. Reproduced with permission from ref (94). Copyright 2017 Nature Publishing Ltd.

In 2017, the work by Jelic et al. unveiled the subpicosecond scale electron tunneling process between the Si(111)-(7×7) surface states and the bulk states of Si.^94^ In this study, the free-space THz pulses with a strong electric field (−200 V/cm) could induce a tunneling current that depended exponentially on tip–sample separation at the STM tunnel junction (Figure 5b). Under illumination with single-cycle THz pulses (Figure 5a), the spatial mapping of the Si(111)-(7×7) surface at the constant-current mode without a DC bias could resolve the Si adatoms and well reproduce the conventional STM topographic image taken over the same area. The agreement in image appearance suggests the localized nature of the THz-driven tunneling electrons. The subpicosecond time window for electron tunneling was revealed in the autocorrelation measurement with paired identical but weaker THz pulses (−100 V/cm), which are not strong enough to induce a detectable current individually (Figure 6e). Surprisingly, an extreme field-induced transient current of ∼160 μA through the Si(111)-(7×7) surface was derived from the −20 pA tunneling current set point used in the THz constant-current imaging after the width and repetition rate of the THz pulses were taken into account. This unusually large current was attributed to the opening of additional tunneling channels between the surface and bulk states. The authors suggested that a large amount of THz-induced tunneling electrons into (or from) the Si(111)-(7×7) transiently saturate (Figure 5c) (or deplete) (Figure 5d) the surface states because of the poor lateral conductivity, which consequentially splits the originally aligned Fermi levels of the bulk and surface and introduces a new tunneling pathway. This mechanism was supported by the observation of faulted–unfaulted asymmetry (Figure 5e and Figure 6a,c,d) in the unit cell taken with either a negative (Figure 6c) or positive (Figure 6d) THz field. Such a topographic asymmetry is usually absent in conventional STM images taken with a positive bias (Figure 6b). However, in the case of THz-induced tunneling, since the surface states leading to the faulted–unfaulted asymmetry are close to the surface Fermi level, they always contribute to the electron tunneling, either from the surface to the tip at the negative half cycle (Figure 5d) of the THz pulse or from the surface to the bulk at the positive half cycle (Figure 5c). The unusual electron tunneling pathway revealed in this study and the large THz-induced current potentially provide a new strategy in fabricating THz electro-optical functional devices.

**Figure 7:**
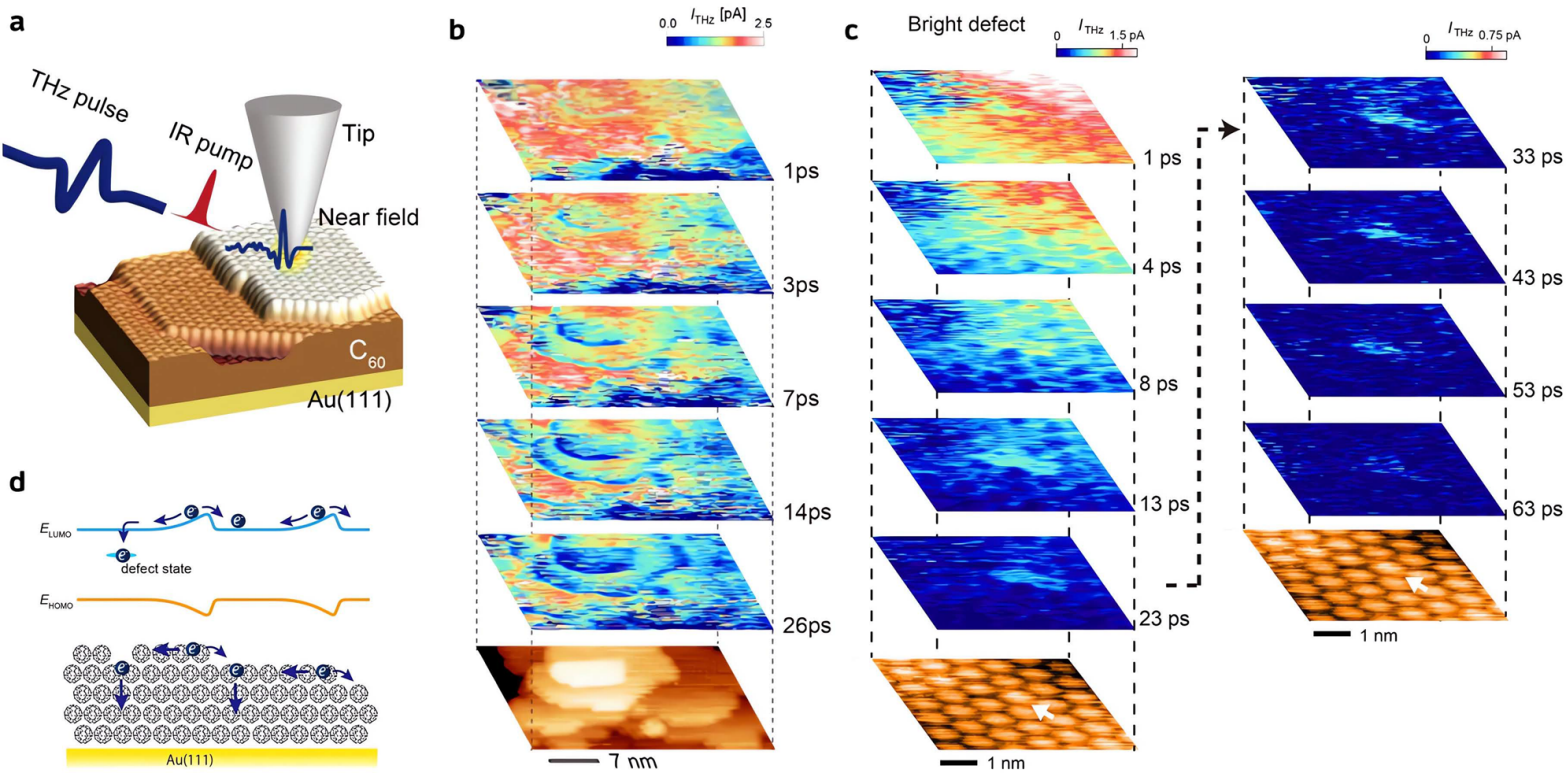
Nanoscale ultrafast electron diffusion in C_60_ thin films on Au(111). (a) Schematic of the experimental setup of the pump–probe STM. (b) Spatial mapping of THz-induced tunneling current at different delay times over several C_60_ terraces, as indicated by the bottom STM image. (c) Spatial mapping of THz-induced tunneling current at different delay times over an area with a misorientation defect, which is shown by the white arrows. (d) Schematic of the relaxation processes of C_60_ LUMO electrons. Reproduced with permission from ref (95). Copyright 2021 American Chemical Society.

**Figure 8:**
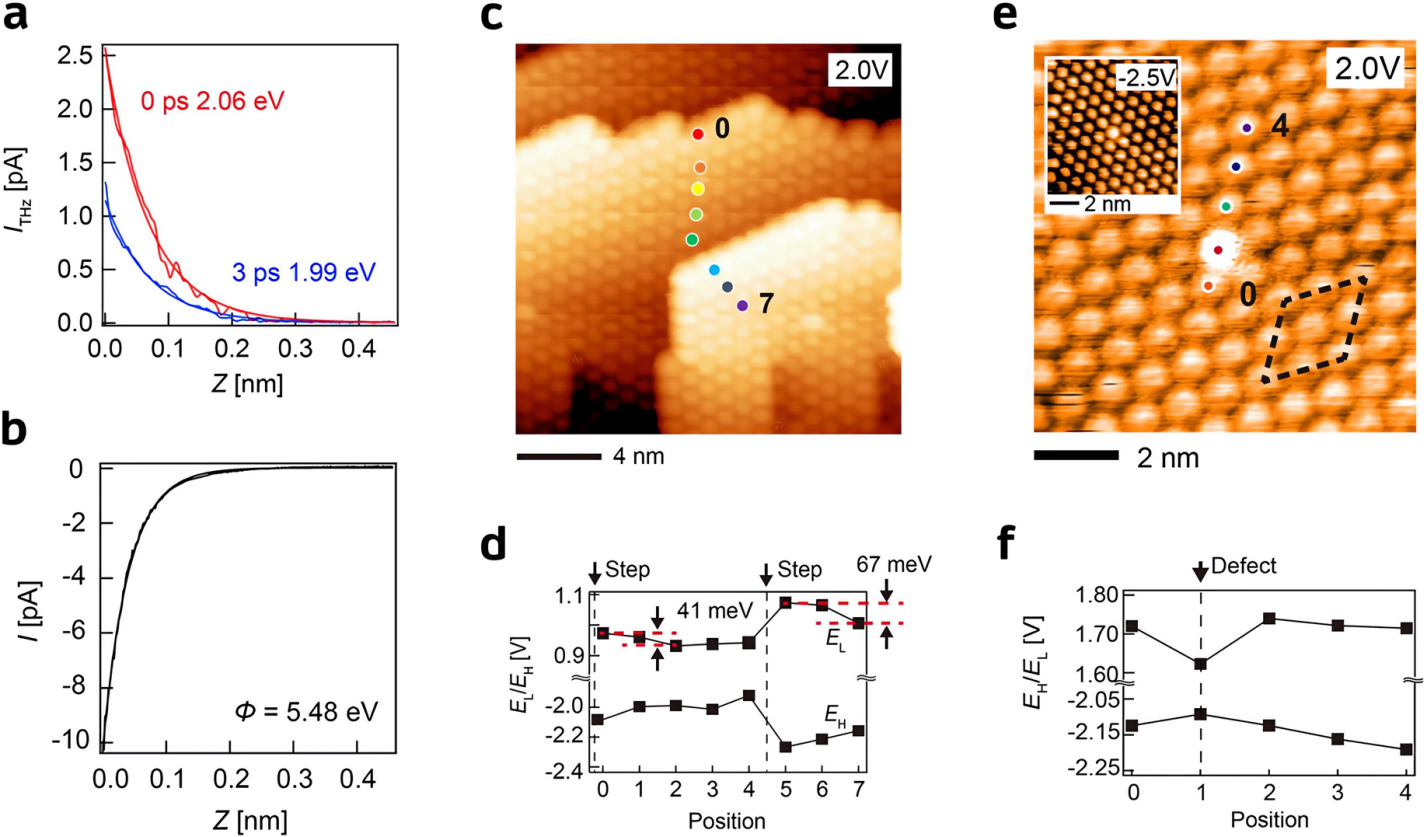
Electronic structure characterization of C_60_ thin films on Au(111). (a) THz-induced tunneling current *I*_THz_ at different delay times and (b) DC tunneling current *I* as a function of the tunneling gap *Z*. The exponential fittings give an apparent tunnel barrier of around 2 eV for *I*_THz_ and 5.48 eV for *I*. (c) STM topographic image of the C_60_ with step edges. (d) The LUMO (*E*_L_) and HOMO (*E*_H_) energies of C_60_ measured at positions indicated in (c) with STS, showing the local minima of *E*_L_ down the step edges. (e) STM topographic image of the C_60_ with a misorientation defect. (f) *E*_L_ and *E*_H_ acquired at locations shown in (e), exhibiting a local minimum of *E*_L_ at the defect site. Reproduced with permission from ref (95). Copyright 2021 American Chemical Society.

Recently, Yoshida et al. visualized the nanometer- and picosecond-scale electron diffusion dynamics in C_60_ thin films on Au(111) and showcased the unique capability of TR-STM to map the spatial inhomogeneity in the ultrafast charge transfer process.^95^ The experimental setup is depicted in Figure 7a, where a 1035 nm, 309 fs laser pulse pumps the Au conduction electrons into the LUMO of C_60_, while a following THz pulse probes the instant population of LUMO electrons by modulating the tunnel barrier and inducing a net tunneling current *I*_THz_. The authors believed that the *I*_THz_ mostly originates from the electrons tunneling from the LUMO of C_60_ to the tip. This mechanism was supported by the ∼2 eV energy barrier of the tunnel junction derived from the *I*_THz_ versus *z* measurement (Figure 8a), which agrees with electron tunneling from the C_60_ LUMO to tip on the basis of the ∼3.1 eV HOMO and LUMO energy gap of C_60_ and the measured 5.48 eV energy barrier with −3 V DC bias where most electrons tunnel from C_60_ HOMO (Figure 8b). The spatial mappings of *I*_THz_ at various delay times clearly show a longer electron trapping time near the lower level of the C_60_ step edge (Figure 7b) and at the defect sites, including both C_60_ vacancy and misorientation (Figure 7c). The scanning tunneling spectroscopy (STS) measurement reveals a nanoscale local energy minimum of LUMO at both the step edge (Figure 8c,d) and the defect sites (Figure 8e,f), either because of the structural discontinuity at the step edge or the formation of in-gap defect states (Figure 7d). Such a local potential minimum is believed to be the origin of the prolonged electron lifetime. The measured *I*_THz_ maximizes across the surveyed areas within 1 ps, followed by a gradual decay in several tens of picoseconds, which indicates that horizontal electron diffusion occurs much faster than the vertical relaxation back into the Au substrate (Figure 7b,c). The horizontal charge redistribution process might be resolved in the future with a shorter laser pulse.

The electronic dynamics related to surface plasmons resonance often occur within a few femtoseconds and, therefore, require a higher time resolution to visualize. Garg et al. compressed the pulse width of near-infrared pulses to less than 6 fs and succeeded in resolving the very fast collective electron oscillation and its decaying process with STM.^96^ In this experiment, localized surface plasmon resonances (LSPRs) in a Au nanorod on n-doped 6H-SiC were excited with CEP-stable pump laser pulses. The probe pulses delayed by a time Δ*t* triggered the electron tunneling from the Au to the tip. The tunneling current from the pump–probe measurement oscillated at a period of ∼2.5 fs, which corresponded to the LSPR with an ∼750 nm wavelength. The light-induced current signal decayed in ∼40 fs, which was assigned to the decay of the LSPR because of the electron–electron scattering. This study demonstrated the capability of using TR-STM to study ultrafast electronic dynamics at the attosecond scale.

Guo et al. applied the TR-STM to investigate the dynamics of electrons captured by the surface oxygen vacancies (V_o_) on rutile TiO_2_(110).^97^ The STS measurement identified an in-gap defect state localized around V_o_. The authors suggested that polarons form near the V_o_ because of the transfer of the excess electrons trapped by the V_o_ to the nearby Ti to form Ti^3+^ ions, which is supported by density-functional theory calculations. The pump–probe measurement with paired nanosecond 532 nm laser pulses revealed an exponentially decaying photon-induced tunneling current (*I*_ph_) close to a V_o_ site, which was assigned to the retrapping dynamics of the photoexcited electrons from the polaron states. The *I*_ph_ measured between two V_o_ defects decayed much faster, which was attributed to the higher trapping efficiency because of the extra V_o_.

### 3.3 Spin Dynamics

As a natural binary system, electron spin in a solid-state environment is of both scientific and industrial interest for its potential application as the one of the building blocks for quantum computation and quantum information.^129−133^ The spin lifetime and dephasing time, two important parameters for assessing the quality of the qubit, are susceptible to the local environment, such as the impurities nearby and the electron bath.^134−136^ Therefore, it is important to investigate the spin relaxation and dephasing processes at the spatial limit. In the following section, we will summarize the recent advancements of TR-STM on the spin dynamics in atomic/molecular magnets and semiconductors.

The groundbreaking experiment by Loth et al. in 2010 first revealed the atomic-scale electron spin relaxation dynamics.^98^ In this experiment, they excited the spin state of a Fe–Cu dimer on the CuN/Cu(100) in an external magnetic field with a strong pump voltage pulse. The evolving spin state was sequentially read out by detecting the spin-polarized current generated by a weaker probe voltage pulse after a pump–probe delay time Δ*t*. The spin relaxation lifetime was extracted to be ∼87 ns from the exponential decay of the spin-polarized current as a function of Δ*t*. In the following decade, this all-electronic pump–probe scheme was extended to the detection and control of the spin dynamics of few-atom/molecule systems. In 2015, Yan et al. succeeded in manipulating the microsecond-scale spin lifetime of a linear antiferromagnetic Fe trimer on Cu_2_N/Cu(100) in an external magnetic field with the Heisenberg exchange interaction.^101^ They found that the exchange interaction between the paramagnetic tip and Fe atoms modifies the state mixing of the two lowest-lying spin states of the Fe trimer as an effective magnetic field and, hence, either increases or decreases the lifetime of the excited higher-lying spin state depending on the antiferromagnetic or ferromagnetic alignment of the magnetic moments between the tip and Fe atoms. In 2017, Paul et al. showcased the control over the millisecond-scale spin lifetime of single Fe atoms on MgO/Ag(001).^137^ Since the spin relaxes through the exchange of energy and angular momentum with either the tip electrons or the metal substrate electrons, the spin lifetime varies in response to the tip-Fe distance and the thickness of MgO, which both influence the population of electrons available for inelastic scattering with the excited spin. More recently, the integration of all-electronic pump–probe STM with the electron spin resonance technique has allowed the characterization and manipulation of the coherent spin evolution in several atomic^40,42,43,102^ and molecular^138^ systems.

**Figure 9:**
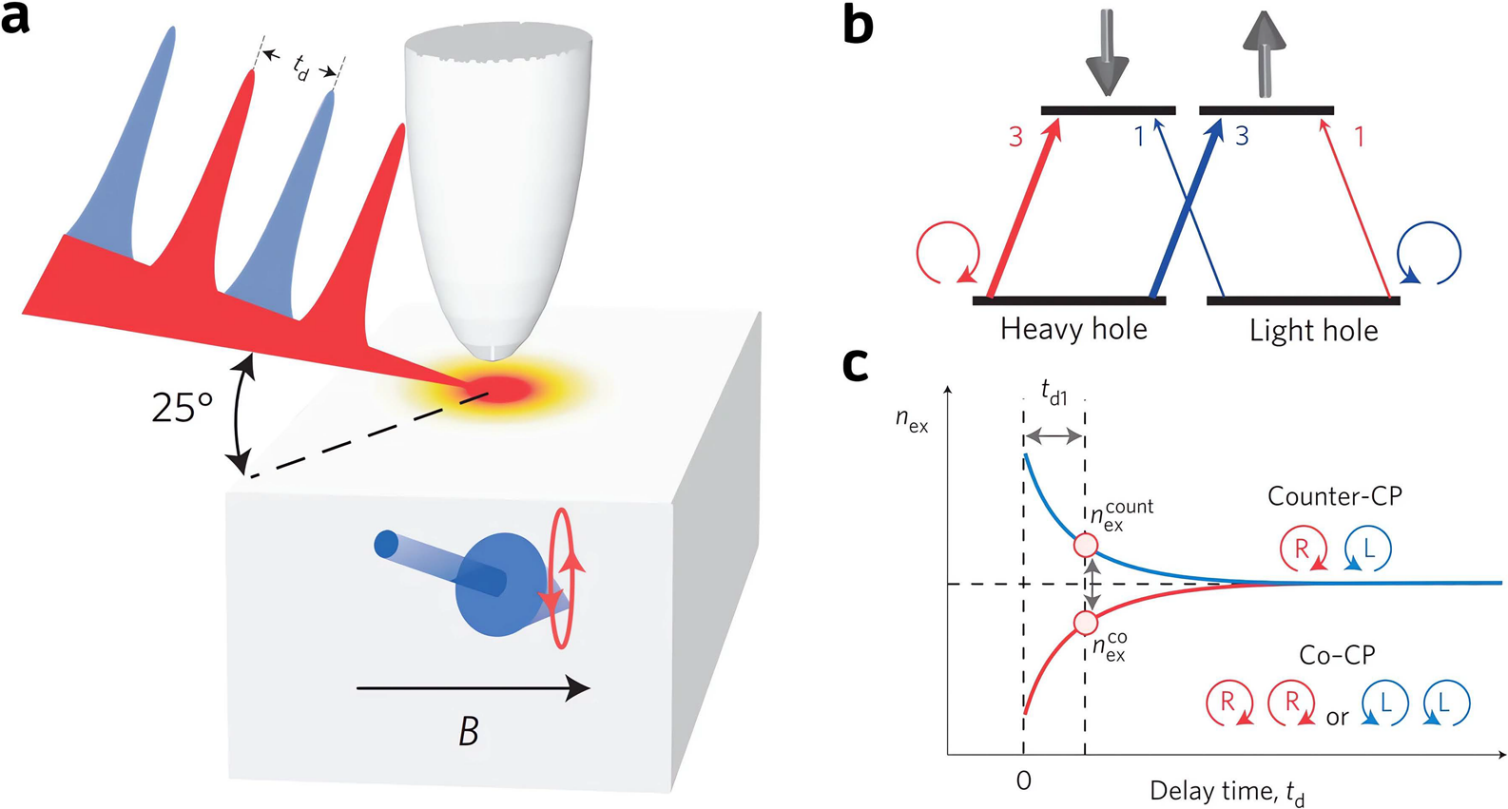
Schematic and mechanism of detecting spin dynamics in GaAs with vis/NIR pump–probe STM. (a) Schematic of detecting spin precession in GaAs with the vis/NIR pump–probe STM. (b) Illustration of transition from heavy- or light-hole bands to conduction band of GaAs under excitation by circularly polarized light. (c) The number of electrons excited with counter-CP (*n*_ex_^count^) or co-CP (*n*_ex_^co^) mode as a function of delay time. Reproduced with permission from ref (99). Copyright 2014 Nature Publishing Ltd.

The temporal resolution of the STM pump–probe spectroscopy with electronic pulses remains limited to nanoseconds, thereby restricting its applications on capturing faster spin dynamics, such as the processes involving the strong spin–orbit coupling (SOC) in semiconductors. Alternatively, circularly polarized (CP) light carries an angular momentum that can alternate the spin state and, therefore, be used to probe the fast spin processes. In 2014, Yoshida et al. extended the vis/NIR pump–probe measurement with STM to the spin relaxation and precession processes in GaAs.^99^ In GaAs, photo absorption of either +1 or −1 spin angular momentum induces a 50% spin polarization in the conduction band because of the characteristic 3:1 ratio between the photoexcited electrons from the *m*_s_ = ±3/2 heavy-hole and *m*_s_ = ±1/2 light-hole valence bands (Figure 9b). The population decay and the precession of the spin-polarized electrons excited by a CP pump NIR pulse can be detected by a probe CP NIR pulse after a delay time of *t*_d_. The handedness of the pump and probe pulses can be either the same or opposite, which is referred to as co-CP and counter-CP excitation, respectively. The co-CP mode generates a lower photoinduced current than the counter-CP mode at a small delay time because of the depletion of the valence band electrons by the pump pulse (Figure 9c). In the experiment, the authors modulated the polarization of the 90 MHz NIR pump/probe pulse trains with a pocket cell and one waveplate to generate a series of alternating co-CP and counter-CP pulse pairs. The difference between the tunneling current induced by the co-CP and counter-CP excitations (Δ*I*_S_) was used to characterize the spin dynamics.

**Figure 10:**
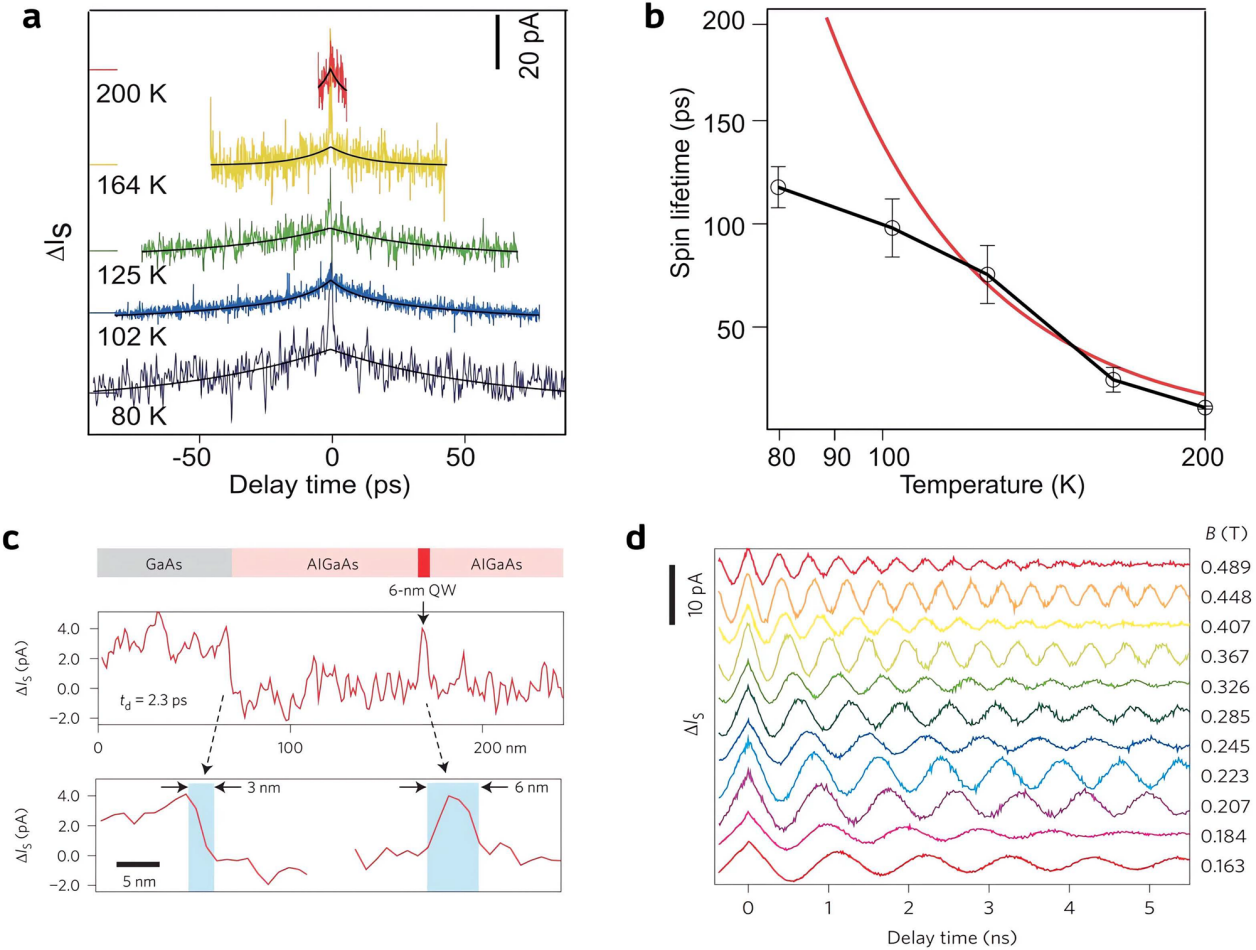
Spin relaxation and precession in GaAs with vis/NIR pump–probe STM. (a) Δ*I*_S_ measured over a p-type GaAs(110) as a function of delay time under different temperatures. (b) Spin lifetime extracted from (d) as a function of temperature, which is fit with *A* × *T*^–3^ (red curve) at the high-temperature side. (c) Δ*I*_S_ across the surface area in the top panel at a delay time of 2.3 ps, showing the joint nm–ps resolution. (d) Oscillatory features of Δ*I*_S_ taken over an n-type GaAs with various in-plane magnetic fields. Reproduced with permission from ref (99). Copyright 2014 Nature Publishing Ltd.

Yoshida et al. studied the spin dynamics in three different GaAs samples.^99^ First of all, they reported the temperature-dependent spin relaxation in p-type GaAs(110) as a proof-of-concept experiment. The spin lifetime (τ_S_) at five different temperatures was extracted by fitting Δ*I*_S_ as a function of *t*_d_ (Figure 10a). The dominant relaxation channel through scattering by impurities at a high temperature (Dyakonov–Perel mechanism) is confirmed by the relationship of τ_S_ = ∼*T*^–3^, while τ_*S*_ is limited by the electron–hole coupling (Bir–Aronov–Pikus mechanism) in the hole-doped GaAs, as shown by the tendency of saturation at a low temperature (Figure 10b). In the following measurement, they showcased the joint spatial–temporal resolution on GaAs/AlGaAs quantum wells at room temperature. The measured Δ*I*_S_ at *t*_d_ = 2.3 ps increased both near the GaAs/AlGaAs interface and inside the 6 nm quantum well, thereby demonstrating a ps–nm resolution across the sample surface (Figure 10c). The τ_S_ obtained at the 6 nm (τ_S_ = 68 ± 6 ps) and 8 nm (τ_S_ = 112 ± 6 ps) wide quantum wells agreed with the Dyakonov–Perel mechanism where the τ_S_ relates to the width-dependent confinement energy *E*_1e_ as τ_S_ = ∼*E*_1e_^–2^. Last but not least, the pump–probe measurement on the n-type GaAs in an external magnetic field at 2.5 K revealed the coherent dynamics of spin. As shown in Figure 10d, the measured Δ*I*_S_ exhibits a clear oscillation whose frequency depends on the magnitude of the external magnetic fields. These oscillations serve as the signatures of the spin precession of the conduction band electrons. Here, although not explicitly stated by the original authors, we attribute the mechanism of the measured oscillations in Δ*I*_S_ to the magneto-optic Kerr effect. In the classical picture, the Larmor procession of the spin excited by the pump pulse leads to a time-dependent spin magnetic moment rotating around the magnetic field direction. This alternating magnetic moment effectively changes the absorption of the probe pulse and, therefore, leads to an oscillatory Δ*I*_S_ following the pace of the spin procession.

## 4 Conclusions and Outlook

In this review, we have discussed the recent application of TR-STM in the studies of ultrafast dynamics beyond the typical electronic response time. The STM gains a temporal resolution at different time scales from its union with short pulses of electrons, THz wave, and/or vis/NIR photons. The usage of short electron pulses allows us to resolve the activities at the order of nanoseconds and has been applied to investigate the spin dynamics. The introduction of free-space THz pulses further brings the resolution down to the subpicosecond scale, which can catch many of the electronic and vibrational dynamics. The use of ultrashort vis/NIR laser pulses can not only provide finer femtosecond scale details of the abovementioned dynamics but also enable the atomic-scale study of even faster processes, such as the excitation and relaxation of surface plasmons. Furthermore, time-resolved TERS opens new avenues of tracing and control of coherence, thereby groundbreakingly expanding the potential applications of TR-STM. These advances have allowed for the nanoscale visualization of many nonequilibrium states, as well as their response to the local inhomogeneous environment. The energy, intensity, polarization, and phases of the driving pulses serve as a series of easily accessible tuning knobs for us to access and control these transient processes at the atomic and molecular levels.

The TR-STM provided a new window to view the transient motion of the nucleus from the stretching of a single molecular bond to the collective motion of atoms in a lattice. A THz pulse focused at the tunneling junction can excite the vibrations in small and large molecules by distinct mechanisms from the transient ionization of the molecule to the impulsive force because of the focused electric field near the tip. It can also generate standing acoustic phonon waves in thin films. These nuclear dynamics can also be triggered by either the direct adsorption of infrared photons or by the Raman scattering of visible or NIR light. The temporal evolution of these processes is traced by either the photoinduced current or the transition rate of a molecular reaction. The measured lifetime ranges from picoseconds of individual molecules adsorbed on conductors to several hundred picoseconds for phonons in a thin metal film. Many of these processes exhibit dramatic variation in response to even saddle changes in the local environment, such as the variations in van der Waals forces between adsorbates and the substrate or the Coulomb interaction between molecules, which is an inaccessible area for other experimental approaches without the simultaneous spatial and temporal resolution.

The TR-STM has also shed light on the charge carrier dynamics in systems ranging from a single dopant or defect in a semiconductor to LSPRs confined near the tip and a conducting substrate. The THz pulses impinging on the organic or inorganic semiconductors can alter the band bending near the surface, thereby leading to nonequilibrium electron tunneling occurring within a picosecond. The local detection of THz-induced current signal with STM helps discriminate the spatial inhomogeneous dynamics resulting from local structural disorders. At the attosecond time scale, the ultrashort CEP-stable NIR pules can stimulate and detect the collective electron oscillation whose oscillation direction depends on the polarity of the pump pulse. These studies have deepened our understanding of how the local electronic or chemical structures can change the lifetime of excited charge carriers in different materials that serve as guidance for the future design of optical electro devices.

The spin dynamics of the charge carriers in solid-state materials or an isolated magnetic surface impurity can be excited and traced using electron or photon pulses. Photons with different angular momentum can control the spin orientation of the conducting electron in semiconductors. The transitions between spin levels split by an external magnetic field can also be excited by electron pulses. Manipulation of the spin population can be achieved by adjusting either the pulse intensity or duration. The characterization and manipulation of the spin degree of freedom in both space and time domains are especially impactful in quantum information science, where spin-based quantum sensing or computing algorithms have exhibited advantages compared with their classical counterparts.

Besides the demonstrated success in the aforementioned areas, TR-STM promises to make unique contributions in many other fields in physics, chemistry, and quantum information science. As a versatile approach, we expect that it readily adapts itself to the investigation of emerging materials. Among others, low-dimensional van der Waals heterostructures have exhibited rich and exotic physics originating from the nonequilibrium charge carriers.^139−143^ The TR-STM can visualize the excitation, spatial distribution, and temporal decay of these charge carriers to provide unparalleled, detailed, and comprehensive information in both space and time. In chemistry, this new technique can broaden our knowledge of nanocatalysis where the interplay between local quantum confinement effects and the chemical transition pathway can be visualized for the first time. Moreover, the coherent manipulation of light–matter interactions can now occur with atomic accuracy,^144^ which may provide an alternate approach to switch the quantum state population of a qubit. Even though TR-STM is currently a sophisticated technique only available in a limited number of research laboratories, recent advances in technology have made TR-STM more accessible and user-friendly.^92,97,106,108,109,145−152^ Because of its versatility, we highly anticipate its future as a widely used technique that is accessible to users in a diverse array of fields.

## References

[ref1] Binnig G.; Rohrer H.; Gerber C.; Weibel E. Tunneling through a controllable vacuum gap. Appl. Phys. Lett. 1982, 40 (2), 178–180. 10.1063/1.92999.

[ref2] Gross L.; Mohn F.; Moll N.; Liljeroth P.; Meyer G. The chemical structure of a molecule resolved by atomic force microscopy. Science 2009, 325 (5944), 1110–1114. 10.1126/science.1176210. 19713523

[ref3] Gross L.; Mohn F.; Moll N.; Meyer G. A.; Ebel R.; Abdel-Mageed W. M.; Jaspars M. Organic structure determination using atomic-resolution scanning probe microscopy. Nature Chem. 2010, 2 (10), 821–825. 10.1038/nchem.765. 20861896

[ref4] Gross L.; Mohn F.; Moll N.; Schuler B.; Criado A.; Guitián E.; Peña D.; Gourdon A.; Meyer G. A. Bond-Order Discrimination by Atomic Force Microscopy. Science 2012, 337, 1326–1329. 10.1126/science.1225621. 22984067

[ref5] Zhang J.; Chen P.; Yuan B.; Ji W.; Cheng Z.; Qiu X. Real-Space Identification of Intermolecular Bonding with Atomic Force Microscopy. Science 2013, 342, 611–614. 10.1126/science.1242603. 24072819

[ref6] Chiang C.-l.; Xu C.; Han Z.; Ho W. Real-space imaging of molecular structure and chemical bonding by single-molecule inelastic tunneling probe. Science 2014, 344 (6186), 885–888. 10.1126/science.1253405. 24855265

[ref7] Pawlak R.; Kisiel M.; Klinovaja J.; Meier T.; Kawai S.; Glatzel T.; Loss D.; Meyer E. Probing Atomic Structure and Majorana Wavefunctions in Mono-Atomic Fe-chains on Superconducting Pb-Surface. arXiv (Atomic and Molecular Clusters), August 21, 2015, 1505.06078, ver. 2. 10.1038/npjqi.2016.35.

[ref8] Schuler B.; Meyer G. A.; Peña D.; Mullins O. C.; Gross L. Unraveling the Molecular Structures of Asphaltenes by Atomic Force Microscopy. J. Am. Chem. Soc. 2015, 137 (31), 9870–9876. 10.1021/jacs.5b04056. 26170086

[ref9] Riss A.; Paz A. P.; Wickenburg S.; Tsai H.-Z.; De Oteyza D. G.; Bradley A. J.; Ugeda M. M.; Gorman P.; Jung H. S.; Crommie M. F.; Rubio A.; Fischer F. R. Imaging single-molecule reaction intermediates stabilized by surface dissipation and entropy. Nature Chem. 2016, 8 (7), 678–683. 10.1038/nchem.2506. 27325094

[ref10] Han Z.; Czap G.; Chiang C.-l.; Xu C.; Wagner P. J.; Wei X.; Zhang Y.; Wu R.; Ho W. Imaging the halogen bond in self-assembled halogenbenzenes on silver. Science 2017, 358, 206–210. 10.1126/science.aai8625. 28912131

[ref11] Gross L.; Schuler B.; Pavliček N.; Fatayer S.; Majzik Z.; Moll N.; Peña D.; Meyer G. Atomic force microscopy for molecular structure elucidation. Angew. Chem., Int. Ed. 2018, 57 (15), 3888–3908. 10.1002/anie.201703509. 29485190

[ref12] Fatayer S.; Albrecht F.; Zhang Y.; Urbonas D.; Peña D.; Moll N.; Gross L. Molecular structure elucidation with charge-state control. Science 2019, 365 (6449), 142–145. 10.1126/science.aax5895. 31296763

[ref13] Giessibl F. J. The qPlus sensor, a powerful core for the atomic force microscope. Review of scientific instruments 2019, 90 (1), 011101 10.1063/1.5052264. 30709191

[ref14] Peng J.; Sokolov S.; Hernangómez-Pérez D.; Evers F.; Gross L.; Lupton J. M.; Repp J. Atomically resolved single-molecule triplet quenching. Science 2021, 373 (6553), 452–456. 10.1126/science.abh1155. 34437120

[ref15] Gao W.; Kang F.; Qiu X.; Yi Z.; Shang L.; Liu M.; Qiu X.; Luo Y.; Xu W. On-Surface Debromination of C6Br6: C6 Ring versus C6 Chain. ACS Nano 2022, 16 (4), 6578–6584. 10.1021/acsnano.2c00945. 35377612

[ref16] Tersoff; Hamann Theory of the scanning tunneling microscope. Physical review. B, Condensed matter 1985, 31 (2), 805–813. 10.1103/PhysRevB.31.805. 9935822

[ref17] Giannozzi P.; Baroni S.; Bonini N.; Calandra M.; Car R.; Cavazzoni C.; Ceresoli D.; Chiarotti G.; Cococcioni M.; Dabo I.; Dal Corso A.; de Gironcoli S.; Fabris S.; Fratesi G.; Gebauer R.; Gerstmann U.; Gougoussis C.; Kokalj A.; Lazzeri M.; Martin-Samos L.; Marzari N.; Mauri F.; Mazzarello R.; Paolini S.; Pasquarello A.; Paulatto L.; Sbraccia C.; Scandolo S.; Sclauzero G.; Seitsonen A. P.; Smogunov A.; Umari P.; Wentzcovitch R. M. QUANTUM ESPRESSO: a modular and open-source software project for quantum simulations of materials. J. Phys.: Condens. Matter 2009, 21, 395502 10.1088/0953-8984/21/39/395502. 21832390

[ref18] Freysoldt C.; Grabowski B.; Hickel T.; Neugebauer J.; Kresse G.; Janotti A.; Walle C. G. v. d. First-principles calculations for point defects in solids. Rev. Mod. Phys. 2014, 86, 253–305. 10.1103/RevModPhys.86.253.

[ref19] Hjorth Larsen A.; Jørgen Mortensen J.; Blomqvist J.; Castelli I. E.; Christensen R.; Dulak M.; Friis J.; Groves M. N.; Hammer B.; Hargus C.; Hermes E. D.; Jennings P. C.; Bjerre Jensen P.; Kermode J. R.; Kitchin J. R.; Leonhard Kolsbjerg E.; Kubal J.; Kaasbjerg K.; Lysgaard S.; Bergmann Maronsson J.; Maxson T.; Olsen T.; Pastewka L.; Peterson A. A.; Rostgaard C.; Schiøtz J.; Schütt O.; Strange M.; Thygesen K. S.; Vegge T.; Vilhelmsen L. B.; Walter M.; Zeng Z.; Jacobsen K. W. The atomic simulation environment-a Python library for working with atoms. J. Phys.: Condens. Matter 2017, 29 (27), 273002 10.1088/1361-648X/aa680e. 28323250

[ref20] Repp J.; Meyer G.; Stojković S. M.; Gourdon A.; Joachim C. Molecules on Insulating Films: Scanning-Tunneling Microscopy Imaging of Individual Molecular Orbitals. Phys. Rev. Lett. 2005, 94 (2), 026803 10.1103/PhysRevLett.94.026803. 15698209

[ref21] Hahn J.; Lee H.; Ho W. Electronic resonance and symmetry in single-molecule inelastic electron tunneling. Phys. Rev. Lett. 2000, 85 (9), 1914 10.1103/PhysRevLett.85.1914. 10970646

[ref22] Stöckle R. M.; Suh Y. D.; Deckert V.; Zenobi R. Nanoscale chemical analysis by tip-enhanced Raman spectroscopy. Chem. Phys. Lett. 2000, 318 (1–3), 131–136. 10.1016/S0009-2614(99)01451-7.

[ref23] Okabayashi N.; Konda Y.; Komeda T. Inelastic electron tunneling spectroscopy of an alkanethiol self-assembled monolayer using scanning tunneling microscopy. Physical review letters 2008, 100 (21), 217801 10.1103/PhysRevLett.100.217801. 18518634

[ref24] Motobayashi K.; Kim Y.; Ueba H.; Kawai M. Insight into action spectroscopy for single molecule motion and reactions through inelastic electron tunneling. Physical review letters 2010, 105 (7), 076101 10.1103/PhysRevLett.105.076101. 20868059

[ref25] Zhang R.; Zhang Y.; Dong Z. C.; Jiang S.; Zhang C.; Chen L. G.; Zhang L.; Liao Y.; Aizpurua J.; Luo Y.; Yang J. L.; Hou J. G. Chemical mapping of a single molecule by plasmon-enhanced Raman scattering. Nature 2013, 498 (7452), 82–86. 10.1038/nature12151. 23739426

[ref26] Jiang S.; Zhang Y.; Zhang R.; Hu C.; Liao M.; Luo Y.; Yang J.; Dong Z.; Hou J. Distinguishing adjacent molecules on a surface using plasmon-enhanced Raman scattering. Nature Nanotechnol. 2015, 10 (10), 865–869. 10.1038/nnano.2015.170. 26214250

[ref27] Guo J.; Lu J.-T.; Feng Y.; Chen J.; Peng J.; Lin Z.; Meng X.; Wang Z.; Li X.-Z.; Wang E.-G.; Jiang Y. Nuclear quantum effects of hydrogen bonds probed by tip-enhanced inelastic electron tunneling. Science 2016, 352 (6283), 321–325. 10.1126/science.aaf2042. 27081066

[ref28] Liao M.; Jiang S.; Hu C.; Zhang R.; Kuang Y.; Zhu J.; Zhang Y.; Dong Z. Tip-enhanced Raman spectroscopic imaging of individual carbon nanotubes with subnanometer resolution. Nano Lett. 2016, 16 (7), 4040–4046. 10.1021/acs.nanolett.6b00533. 27348072

[ref29] Xu C.; Chiang C.-l.; Han Z.; Ho W. Nature of asymmetry in the vibrational line shape of single-molecule inelastic electron tunneling spectroscopy with the STM. Phys. Rev. Lett. 2016, 116 (16), 166101 10.1103/PhysRevLett.116.166101. 27152811

[ref30] Czap G.; Wagner P. J.; Xue F.; Gu L.; Li J.; Yao J.; Wu R.; Ho W. Probing and imaging spin interactions with a magnetic single-molecule sensor. Science 2019, 364 (6441), 670–673. 10.1126/science.aaw7505. 31097665

[ref31] Stroscio J. A.; Eigler D. M. Atomic and Molecular Manipulation with the Scanning Tunneling Microscope. Science 1991, 254 (5036), 1319–1326. 10.1126/science.254.5036.1319. 17773601

[ref32] Eigler D. M.; Schweizer E. K. Positioning single atoms with a scanning tunnelling microscope. Nature 1990, 344 (6266), 524–526. 10.1038/344524a0.

[ref33] Stroscio J. A.; Celotta R. J. Controlling the Dynamics of a Single Atom in Lateral Atom Manipulation. Science 2004, 306 (5694), 242–247. 10.1126/science.1102370. 15358867

[ref34] Kudernac T.; Ruangsupapichat N.; Parschau M.; Maciá B.; Katsonis N.; Harutyunyan S. R.; Ernst K.-H.; Feringa B. L. Electrically driven directional motion of a four-wheeled molecule on a metal surface. Nature 2011, 479 (7372), 208–211. 10.1038/nature10587. 22071765

[ref35] Grill L.; Dyer M.; Lafferentz L.; Persson M.; Peters M. V.; Hecht S. Nano-architectures by covalent assembly of molecular building blocks. Nat. Nanotechnol. 2007, 2 (11), 687–691. 10.1038/nnano.2007.346. 18654406

[ref36] Emmrich M.; Schneiderbauer M.; Huber F.; Weymouth A. J.; Okabayashi N.; Giessibl F. J. Force Field Analysis Suggests a Lowering of Diffusion Barriers in Atomic Manipulation Due to Presence of STM Tip. Phys. Rev. Lett. 2015, 114 (14), 146101 10.1103/PhysRevLett.114.146101. 25910137

[ref37] Li S.; Czap G.; Li J.; Zhang Y.; Yu A.; Yuan D.; Kimura H.; Wu R.; Ho W. Confinement-Induced Catalytic Dissociation of Hydrogen Molecules in a Scanning Tunneling Microscope. J. Am. Chem. Soc. 2022, 144 (22), 9618–9623. 10.1021/jacs.2c00005. 35486711

[ref38] Wagner C.; Green M. F. B.; Maiworm M.; Leinen P.; Esat T.; Ferri N.; Friedrich N.; Findeisen R.; Tkatchenko A.; Temirov R.; Tautz F. S. Quantitative imaging of electric surface potentials with single-atom sensitivity. Nat. Mater. 2019, 18 (8), 853–859. 10.1038/s41563-019-0382-8. 31182779 PMC6656579

[ref39] Zhang X.; Wolf C.; Wang Y.; Aubin H.; Bilgeri T.; Willke P.; Heinrich A. J.; Choi T. Electron spin resonance of single iron phthalocyanine molecules and role of their non-localized spins in magnetic interactions. Nat. Chem. 2022, 14 (1), 59–65. 10.1038/s41557-021-00827-7. 34764471

[ref40] Bae Y.; Yang K.; Willke P.; Choi T.; Heinrich A. J.; Lutz C. P. Enhanced quantum coherence in exchange coupled spins via singlet-triplet transitions. Science Advances 2018, 4 (11), eaau4159 10.1126/sciadv.aau4159. 30430136 PMC6226279

[ref41] Choi T.; Paul W.; Rolf-Pissarczyk S.; Macdonald A. J.; Natterer F. D.; Yang K.; Willke P.; Lutz C. P.; Heinrich A. J. Atomic-scale sensing of the magnetic dipolar field from single atoms. Nat. Nanotechnol. 2017, 12 (5), 420–424. 10.1038/nnano.2017.18. 28263962

[ref42] Yang K.; Paul W.; Phark S.-H.; Willke P.; Bae Y.; Choi T.; Esat T.; Ardavan A.; Heinrich A. J.; Lutz C. P. Coherent spin manipulation of individual atoms on a surface. Science 2019, 366 (6464), 509–512. 10.1126/science.aay6779. 31649202

[ref43] Baumann S.; Paul W.; Choi T.; Lutz C. P.; Ardavan A.; Heinrich A. J. Electron paramagnetic resonance of individual atoms on a surface. Science 2015, 350 (6259), 417–420. 10.1126/science.aac8703. 26494753

[ref44] Wiesendanger R.; Güntherodt H. J.; Güntherodt G.; Gambino R. J.; Ruf R. Observation of vacuum tunneling of spin-polarized electrons with the scanning tunneling microscope. Phys. Rev. Lett. 1990, 65 (2), 247–250. 10.1103/PhysRevLett.65.247. 10042590

[ref45] Wachowiak A.; Wiebe J.; Bode M.; Pietzsch O.; Morgenstern M.; Wiesendanger R. Direct Observation of Internal Spin Structure of Magnetic Vortex Cores. Science 2002, 298 (5593), 577–580. 10.1126/science.1075302. 12386329

[ref46] Bode M.; Heide M.; von Bergmann K.; Ferriani P.; Heinze S.; Bihlmayer G.; Kubetzka A.; Pietzsch O.; Blügel S.; Wiesendanger R. Chiral magnetic order at surfaces driven by inversion asymmetry. Nature 2007, 447 (7141), 190–193. 10.1038/nature05802. 17495922

[ref47] Heinze S.; von Bergmann K.; Menzel M.; Brede J.; Kubetzka A.; Wiesendanger R.; Bihlmayer G.; Blügel S. Spontaneous atomic-scale magnetic skyrmion lattice in two dimensions. Nat. Phys. 2011, 7 (9), 713–718. 10.1038/nphys2045.

[ref48] Wortmann D.; Heinze S.; Kurz P.; Bihlmayer G.; Blügel S. Resolving Complex Atomic-Scale Spin Structures by Spin-Polarized Scanning Tunneling Microscopy. Phys. Rev. Lett. 2001, 86 (18), 4132–4135. 10.1103/PhysRevLett.86.4132. 11328113

[ref49] Spinelli A.; Bryant B.; Delgado F.; Fernández-Rossier J.; Otte A. F. Imaging of spin waves in atomically designed nanomagnets. Nat. Mater. 2014, 13 (8), 782–785. 10.1038/nmat4018. 24997736

[ref50] Oka H.; Ignatiev P. A.; Wedekind S.; Rodary G.; Niebergall L.; Stepanyuk V. S.; Sander D.; Kirschner J. Spin-Dependent Quantum Interference Within a Single Magnetic Nanostructure. Science 2010, 327 (5967), 843–846. 10.1126/science.1183224. 20150496

[ref51] Enayat M.; Sun Z.; Singh U. R.; Aluru R.; Schmaus S.; Yaresko A.; Liu Y.; Lin C.; Tsurkan V.; Loidl A.; Deisenhofer J.; Wahl P. Real-space imaging of the atomic-scale magnetic structure of Fe_1+*y*_Te. Science 2014, 345 (6197), 653–656. 10.1126/science.1251682. 25081481

[ref52] González-Herrero H.; Gómez-Rodríguez J. M.; Mallet P.; Moaied M.; Palacios J. J.; Salgado C.; Ugeda M. M.; Veuillen J.-Y.; Yndurain F.; Brihuega I. Atomic-scale control of graphene magnetism by using hydrogen atoms. Science 2016, 352 (6284), 437–441. 10.1126/science.aad8038. 27102478

[ref53] Chen W.; Sun Z.; Wang Z.; Gu L.; Xu X.; Wu S.; Gao C. Direct observation of van der Waals stacking–dependent interlayer magnetism. Science 2019, 366 (6468), 983–987. 10.1126/science.aav1937. 31753996

[ref54] Yin J.-X.; Ma W.; Cochran T. A.; Xu X.; Zhang S. S.; Tien H.-J.; Shumiya N.; Cheng G.; Jiang K.; Lian B.; Song Z.; Chang G.; Belopolski I.; Multer D.; Litskevich M.; Cheng Z.-J.; Yang X. P.; Swidler B.; Zhou H.; Lin H.; Neupert T.; Wang Z.; Yao N.; Chang T.-R.; Jia S.; Zahid Hasan M. Quantum-limit Chern topological magnetism in TbMn6Sn6. Nature 2020, 583 (7817), 533–536. 10.1038/s41586-020-2482-7. 32699400

[ref55] Kamber U.; Bergman A.; Eich A.; Iuşan D.; Steinbrecher M.; Hauptmann N.; Nordström L.; Katsnelson M. I.; Wegner D.; Eriksson O.; Khajetoorians A. A. Self-induced spin glass state in elemental and crystalline neodymium. Science 2020, 368 (6494), eaay6757 10.1126/science.aay6757. 32467362

[ref56] Repicky J.; Wu P.-K.; Liu T.; Corbett J. P.; Zhu T.; Cheng S.; Ahmed A. S.; Takeuchi N.; Guerrero-Sanchez J.; Randeria M.; Kawakami R. K.; Gupta J. A. Atomic-scale visualization of topological spin textures in the chiral magnet MnGe. Science 2021, 374 (6574), 1484–1487. 10.1126/science.abd9225. 34914516

[ref57] Linderoth T. R.; Horch S.; Lægsgaard E.; Stensgaard I.; Besenbacher F. Surface Diffusion of Pt on Pt(110): Arrhenius Behavior of Long Jumps. Phys. Rev. Lett. 1997, 78 (26), 4978–4981. 10.1103/PhysRevLett.78.4978.

[ref58] Wintterlin J.; Trost J.; Renisch S.; Schuster R.; Zambelli T.; Ertl G. Real-time STM observations of atomic equilibrium fluctuations in an adsorbate system: O/Ru(0001). Surf. Sci. 1997, 394 (1), 159–169. 10.1016/S0039-6028(97)00604-3.

[ref59] Rost M. J.; Crama L.; Schakel P.; Tol E. v.; Velzen-Williams G. B. E. M. v.; Overgauw C. F.; Horst H. t.; Dekker H.; Okhuijsen B.; Seynen M.; Vijftigschild A.; Han P.; Katan A. J.; Schoots K.; Schumm R.; Loo W. v.; Oosterkamp T. H.; Frenken J. W. M. Scanning probe microscopes go video rate and beyond. Rev. Sci. Instrum. 2005, 76 (5), 053710 10.1063/1.1915288.

[ref60] Swartzentruber B. S. Direct Measurement of Surface Diffusion Using Atom-Tracking Scanning Tunneling Microscopy. Phys. Rev. Lett. 1996, 76 (3), 459–462. 10.1103/PhysRevLett.76.459. 10061462

[ref61] Borovsky B.; Krueger M.; Ganz E. Piecewise diffusion of the silicon dimer. Phys. Rev. B 1999, 59 (3), 1598–1601. 10.1103/PhysRevB.59.1598.

[ref62] Qin X. R.; Swartzentruber B. S.; Lagally M. G. Diffusional Kinetics of SiGe Dimers on Si(100) Using Atom-Tracking Scanning Tunneling Microscopy. Phys. Rev. Lett. 2000, 85 (17), 3660–3663. 10.1103/PhysRevLett.85.3660. 11030975

[ref63] Hill E.; Freelon B.; Ganz E. Diffusion of hydrogen on the Si(001) surface investigated by STM atom tracking. Phys. Rev. B 1999, 60 (23), 15896–15900. 10.1103/PhysRevB.60.15896.

[ref64] Swartzentruber B. S.; Smith A. P.; Jónsson H. Experimental and Theoretical Study of the Rotation of Si Ad-dimers on the Si(100) Surface. Phys. Rev. Lett. 1996, 77 (12), 2518–2521. 10.1103/PhysRevLett.77.2518. 10061974

[ref65] Schoenlein R. W.; Peteanu L. A.; Mathies R. A.; Shank C. V. The First Step in Vision: Femtosecond Isomerization of Rhodopsin. Science 1991, 254 (5030), 412–415. 10.1126/science.1925597. 1925597

[ref66] Kim J. E.; McCamant D. W.; Zhu L.; Mathies R. A. Resonance Raman Structural Evidence that the Cis-to-Trans Isomerization in Rhodopsin Occurs in Femtoseconds. J. Phys. Chem. B 2001, 105 (6), 1240–1249. 10.1021/jp001236s. 16755302 PMC1473983

[ref67] Zheng J.; Kwak K.; Asbury J.; Chen X.; Piletic I. R.; Fayer M. D. Ultrafast Dynamics of Solute-Solvent Complexation Observed at Thermal Equilibrium in Real Time. Science 2005, 309 (5739), 1338–1343. 10.1126/science.1116213. 16081697

[ref68] Pestov D.; Murawski R. K.; Ariunbold G. O.; Wang X.; Zhi M.; Sokolov A. V.; Sautenkov V. A.; Rostovtsev Y. V.; Dogariu A.; Huang Y.; Scully M. O. Optimizing the Laser-Pulse Configuration for Coherent Raman Spectroscopy. Science 2007, 316 (5822), 265–268. 10.1126/science.1139055. 17431177

[ref69] Fang C.; Frontiera R. R.; Tran R.; Mathies R. A. Mapping GFP structure evolution during proton transfer with femtosecond Raman spectroscopy. Nature 2009, 462 (7270), 200–204. 10.1038/nature08527. 19907490

[ref70] Namboodiri M.; Kazemi M. M.; Zeb Khan T.; Materny A.; Kiefer J. Ultrafast Vibrational Dynamics and Energy Transfer in Imidazolium Ionic Liquids. J. Am. Chem. Soc. 2014, 136 (16), 6136–6141. 10.1021/ja502527y. 24697246

[ref71] Jakowetz A. C.; Böhm M. L.; Sadhanala A.; Huettner S.; Rao A.; Friend R. H. Visualizing excitations at buried heterojunctions in organic semiconductor blends. Nat. Mater. 2017, 16 (5), 551–557. 10.1038/nmat4865. 28218921

[ref72] Sun D.; Aivazian G.; Jones A. M.; Ross J. S.; Yao W.; Cobden D.; Xu X. Ultrafast hot-carrier-dominated photocurrent in graphene. Nat. Nanotechnol. 2012, 7 (2), 114–118. 10.1038/nnano.2011.243. 22245859

[ref73] Jailaubekov A. E.; Willard A. P.; Tritsch J. R.; Chan W.-L.; Sai N.; Gearba R.; Kaake L. G.; Williams K. J.; Leung K.; Rossky P. J.; Zhu X. Y. Hot charge-transfer excitons set the time limit for charge separation at donor/acceptor interfaces in organic photovoltaics. Nat. Mater. 2013, 12 (1), 66–73. 10.1038/nmat3500. 23223125

[ref74] Tielrooij K. J.; Song J. C. W.; Jensen S. A.; Centeno A.; Pesquera A.; Zurutuza Elorza A.; Bonn M.; Levitov L. S.; Koppens F. H. L. Photoexcitation cascade and multiple hot-carrier generation in graphene. Nat. Phys. 2013, 9 (4), 248–252. 10.1038/nphys2564.

[ref75] Marchioro A.; Teuscher J.; Friedrich D.; Kunst M.; van de Krol R.; Moehl T.; Grätzel M.; Moser J.-E. Unravelling the mechanism of photoinduced charge transfer processes in lead iodide perovskite solar cells. Nat. Photonics 2014, 8 (3), 250–255. 10.1038/nphoton.2013.374.

[ref76] Hong X.; Kim J.; Shi S.-F.; Zhang Y.; Jin C.; Sun Y.; Tongay S.; Wu J.; Zhang Y.; Wang F. Ultrafast charge transfer in atomically thin MoS2/WS2 heterostructures. Nat. Nanotechnol. 2014, 9 (9), 682–686. 10.1038/nnano.2014.167. 25150718

[ref77] Shi D.; Adinolfi V.; Comin R.; Yuan M.; Alarousu E.; Buin A.; Chen Y.; Hoogland S.; Rothenberger A.; Katsiev K.; Losovyj Y.; Zhang X.; Dowben P. A.; Mohammed O. F.; Sargent E. H.; Bakr O. M. Low trap-state density and long carrier diffusion in organolead trihalide perovskite single crystals. Science 2015, 347 (6221), 519–522. 10.1126/science.aaa2725. 25635092

[ref78] Maialle M. Z.; de Andrada e Silva E. A.; Sham L. J. Exciton spin dynamics in quantum wells. Phys. Rev. B 1993, 47 (23), 15776–15788. 10.1103/PhysRevB.47.15776. 10005974

[ref79] Gupta J. A.; Knobel R.; Samarth N.; Awschalom D. D. Ultrafast Manipulation of Electron Spin Coherence. Science 2001, 292 (5526), 2458–2461. 10.1126/science.1061169. 11431559

[ref80] Berezovsky J.; Mikkelsen M. H.; Stoltz N. G.; Coldren L. A.; Awschalom D. D. Picosecond Coherent Optical Manipulation of a Single Electron Spin in a Quantum Dot. Science 2008, 320 (5874), 349–352. 10.1126/science.1154798. 18420929

[ref81] Kim J.; Hong X.; Jin C.; Shi S.-F.; Chang C.-Y. S.; Chiu M.-H.; Li L.-J.; Wang F. Ultrafast generation of pseudo-magnetic field for valley excitons in WSe_2_ monolayers. Science 2014, 346 (6214), 1205–1208. 10.1126/science.1258122. 25477455

[ref82] Sie E. J.; McIver J. W.; Lee Y.-H.; Fu L.; Kong J.; Gedik N. Valley-selective optical Stark effect in monolayer WS2. Nat. Mater. 2015, 14 (3), 290–294. 10.1038/nmat4156. 25502098

[ref83] Xie X.; Doblhoff-Dier K.; Roither S.; Schöffler M. S.; Kartashov D.; Xu H.; Rathje T.; Paulus G. G.; Baltuška A.; Gräfe S.; Kitzler M. Attosecond-Recollision-Controlled Selective Fragmentation of Polyatomic Molecules. Phys. Rev. Lett. 2012, 109 (24), 243001 10.1103/PhysRevLett.109.243001. 23368312

[ref84] Murphy D. B. Fundamentals of light microscopy and electronic imaging; John Wiley & Sons, 2002.

[ref85] Moerner W. E.; Kador L. Optical detection and spectroscopy of single molecules in a solid. Phys. Rev. Lett. 1989, 62 (21), 2535–2538. 10.1103/PhysRevLett.62.2535. 10040013

[ref86] Orrit M.; Bernard J. Single pentacene molecules detected by fluorescence excitation in a p-terphenyl crystal. Phys. Rev. Lett. 1990, 65 (21), 2716–2719. 10.1103/PhysRevLett.65.2716. 10042674

[ref87] Peller D.; Kastner L. Z.; Buchner T.; Roelcke C.; Albrecht F.; Moll N.; Huber R.; Repp J. Sub-cycle atomic-scale forces coherently control a single-molecule switch. Nature 2020, 585 (7823), 58–62. 10.1038/s41586-020-2620-2. 32879499

[ref88] Li S.; Chen S.; Li J.; Wu R.; Ho W. Joint Space-Time Coherent Vibration Driven Conformational Transitions in a Single Molecule. Phys. Rev. Lett. 2017, 119 (17), 176002 10.1103/PhysRevLett.119.176002. 29219451

[ref89] Cocker T. L.; Peller D.; Yu P.; Repp J.; Huber R. Tracking the ultrafast motion of a single molecule by femtosecond orbital imaging. Nature 2016, 539 (7628), 263–267. 10.1038/nature19816. 27830788 PMC5597038

[ref90] Wang L.; Xia Y.; Ho W. Atomic-scale quantum sensing based on the ultrafast coherence of an H2 molecule in an STM cavity. Science 2022, 376 (6591), 401–405. 10.1126/science.abn9220. 35446636

[ref91] Sheng S.; Oeter A.-C.; Abdo M.; Lichtenberg K.; Hentschel M.; Loth S. Launching Coherent Acoustic Phonon Wave Packets with Local Femtosecond Coulomb Forces. Phys. Rev. Lett. 2022, 129 (4), 043001 10.1103/PhysRevLett.129.043001. 35939022

[ref92] Luo Y.; Martin-Jimenez A.; Pisarra M.; Martin F.; Garg M.; Kern K. Imaging and Controlling Coherent Phonon Wave Packets in Single Graphene Nanoribbons. arXiv, October 5, 2022, 2210.02561, ver. 1. 10.48550/arXiv.2210.02561. PMC10264436 37311753

[ref93] Cocker T. L.; Jelic V.; Gupta M.; Molesky S. J.; Burgess J. A. J.; Reyes G. D. L.; Titova L. V.; Tsui Y. Y.; Freeman M. R.; Hegmann F. A. An ultrafast terahertz scanning tunnelling microscope. Nat. Photonics 2013, 7 (8), 620–625. 10.1038/nphoton.2013.151.

[ref94] Jelic V.; Iwaszczuk K.; Nguyen P. H.; Rathje C.; Hornig G. J.; Sharum H. M.; Hoffman J. R.; Freeman M. R.; Hegmann F. A. Ultrafast terahertz control of extreme tunnel currents through single atoms on a silicon surface. Nat. Phys. 2017, 13 (6), 591–598. 10.1038/nphys4047.

[ref95] Yoshida S.; Arashida Y.; Hirori H.; Tachizaki T.; Taninaka A.; Ueno H.; Takeuchi O.; Shigekawa H. Terahertz Scanning Tunneling Microscopy for Visualizing Ultrafast Electron Motion in Nanoscale Potential Variations. ACS Photonics 2021, 8 (1), 315–323. 10.1021/acsphotonics.0c01572.

[ref96] Garg M.; Kern K. Attosecond coherent manipulation of electrons in tunneling microscopy. Science 2020, 367 (6476), 411–415. 10.1126/science.aaz1098. 31727858

[ref97] Guo C.; Meng X.; Fu H.; Wang Q.; Wang H.; Tian Y.; Peng J.; Ma R.; Weng Y.; Meng S.; Wang E.; Jiang Y. Probing Nonequilibrium Dynamics of Photoexcited Polarons on a Metal-Oxide Surface with Atomic Precision. Phys. Rev. Lett. 2020, 124 (20), 206801 10.1103/PhysRevLett.124.206801. 32501065

[ref98] Loth S.; Etzkorn M.; Lutz C. P.; Eigler D. M.; Heinrich A. J. Measurement of Fast Electron Spin Relaxation Times with Atomic Resolution. Science 2010, 329 (5999), 1628–1630. 10.1126/science.1191688. 20929842

[ref99] Yoshida S.; Aizawa Y.; Wang Z.-h.; Oshima R.; Mera Y.; Matsuyama E.; Oigawa H.; Takeuchi O.; Shigekawa H. Probing ultrafast spin dynamics with optical pump–probe scanning tunnelling microscopy. Nat. Nanotechnol. 2014, 9 (8), 588–593. 10.1038/nnano.2014.125. 24974938

[ref100] Natterer F. D.; Donati F.; Patthey F.; Brune H. Thermal and Magnetic-Field Stability of Holmium Single-Atom Magnets. Phys. Rev. Lett. 2018, 121 (2), 027201 10.1103/PhysRevLett.121.027201. 30085712

[ref101] Yan S.; Choi D.-J.; Burgess J. A. J.; Rolf-Pissarczyk S.; Loth S. Control of quantum magnets by atomic exchange bias. Nat. Nanotechnol. 2015, 10 (1), 40–45. 10.1038/nnano.2014.281. 25502311

[ref102] Veldman L. M.; Farinacci L.; Rejali R.; Broekhoven R.; Gobeil J.; Coffey D.; Ternes M.; Otte A. F. Free coherent evolution of a coupled atomic spin system initialized by electron scattering. Science 2021, 372 (6545), 964–968. 10.1126/science.abg8223. 34045351

[ref103] Nunes G.; Freeman M. R. Picosecond Resolution in Scanning Tunneling Microscopy. Science 1993, 262 (5136), 1029–1032. 10.1126/science.262.5136.1029. 17782049

[ref104] Groeneveld R. H. M.; van Kempen H. The capacitive origin of the picosecond electrical transients detected by a photoconductively gated scanning tunneling microscope. Appl. Phys. Lett. 1996, 69 (15), 2294–2296. 10.1063/1.117538.

[ref105] Luo Y.; Jelic V.; Chen G.; Nguyen P. H.; Liu Y.-J. R.; Calzada J. A. M.; Mildenberger D. J.; Hegmann F. A. Nanoscale terahertz STM imaging of a metal surface. Phys. Rev. B 2020, 102 (20), 205417 10.1103/PhysRevB.102.205417.

[ref106] Liu S.; Hammud A.; Hamada I.; Wolf M.; Müller M.; Kumagai T. Nanoscale coherent phonon spectroscopy. Science Advances 2022, 8 (42), eabq5682 10.1126/sciadv.abq5682. 36269832 PMC9586471

[ref107] Terada Y.; Yoshida S.; Takeuchi O.; Shigekawa H. Real-space imaging of transient carrier dynamics by nanoscale pump–probe microscopy. Nat. Photonics 2010, 4 (12), 869–874. 10.1038/nphoton.2010.235.

[ref108] Arashida Y.; Mogi H.; Ishikawa M.; Igarashi I.; Hatanaka A.; Umeda N.; Peng J.; Yoshida S.; Takeuchi O.; Shigekawa H. Subcycle Mid-Infrared Electric-Field-Driven Scanning Tunneling Microscopy with a Time Resolution Higher Than 30 fs. ACS Photonics 2022, 9 (9), 3156–3164. 10.1021/acsphotonics.2c00995.

[ref109] Garg M.; Martin-Jimenez A.; Luo Y.; Kern K. Ultrafast photon-induced tunneling microscopy. ACS Nano 2021, 15 (11), 18071–18084. 10.1021/acsnano.1c06716. 34723474 PMC8613903

[ref110] Garg M.; Martin-Jimenez A.; Pisarra M.; Luo Y.; Martín F.; Kern K. Real-space subfemtosecond imaging of quantum electronic coherences in molecules. Nat. Photonics 2022, 16 (3), 196–202. 10.1038/s41566-021-00929-1.

[ref111] Pozzi E. A.; Sonntag M. D.; Jiang N.; Chiang N.; Seideman T.; Hersam M. C.; Van Duyne R. P. Ultrahigh Vacuum Tip-Enhanced Raman Spectroscopy with Picosecond Excitation. J. Phys. Chem. Lett. 2014, 5 (15), 2657–2661. 10.1021/jz501239z. 26277959

[ref112] Klingsporn J. M.; Sonntag M. D.; Seideman T.; Van Duyne R. P. Tip-Enhanced Raman Spectroscopy with Picosecond Pulses. J. Phys. Chem. Lett. 2014, 5 (1), 106–110. 10.1021/jz4024404. 26276188

[ref113] Luo Y.; Martin-Jimenez A.; Gutzler R.; Garg M.; Kern K. Ultrashort Pulse Excited Tip-Enhanced Raman Spectroscopy in Molecules. Nano Lett. 2022, 22 (13), 5100–5106. 10.1021/acs.nanolett.2c00485. 35704454 PMC9284611

[ref114] Jiang N.; Foley E. T.; Klingsporn J. M.; Sonntag M. D.; Valley N. A.; Dieringer J. A.; Seideman T.; Schatz G. C.; Hersam M. C.; Van Duyne R. P. Observation of Multiple Vibrational Modes in Ultrahigh Vacuum Tip-Enhanced Raman Spectroscopy Combined with Molecular-Resolution Scanning Tunneling Microscopy. Nano Lett. 2012, 12 (10), 5061–5067. 10.1021/nl2039925. 22200250

[ref115] Jaculbia R. B.; Imada H.; Miwa K.; Iwasa T.; Takenaka M.; Yang B.; Kazuma E.; Hayazawa N.; Taketsugu T.; Kim Y. Single-molecule resonance Raman effect in a plasmonic nanocavity. Nat. Nanotechnol. 2020, 15 (2), 105–110. 10.1038/s41565-019-0614-8. 31959928

[ref116] van Schrojenstein Lantman E. M.; Deckert-Gaudig T.; Mank A. J. G.; Deckert V.; Weckhuysen B. M. Catalytic processes monitored at the nanoscale with tip-enhanced Raman spectroscopy. Nat. Nanotechnol. 2012, 7 (9), 583–586. 10.1038/nnano.2012.131. 22902959

[ref117] Li Y.; Wang J.; Clark M. L.; Kubiak C. P.; Xiong W. Characterizing interstate vibrational coherent dynamics of surface adsorbed catalysts by fourth-order 3D SFG spectroscopy. Chem. Phys. Lett. 2016, 650, 1–6. 10.1016/j.cplett.2016.02.031.

[ref118] Jansen T. l. C.; Saito S.; Jeon J.; Cho M. Theory of coherent two-dimensional vibrational spectroscopy. J. Chem. Phys. 2019, 150 (10), 100901 10.1063/1.5083966. 30876372

[ref119] Yoshioka K.; Katayama I.; Minami Y.; Kitajima M.; Yoshida S.; Shigekawa H.; Takeda J. Real-space coherent manipulation of electrons in a single tunnel junction by single-cycle terahertz electric fields. Nat. Photonics 2016, 10 (12), 762–765. 10.1038/nphoton.2016.205.

[ref120] Kim C. H.; Joo T. Coherent excited state intramolecular proton transfer probed by time-resolved fluorescence. Phys. Chem. Chem. Phys. 2009, 11 (44), 10266–10269. 10.1039/b915768a. 19890507

[ref121] Meng X.; Guo J.; Peng J.; Chen J.; Wang Z.; Shi J.-R.; Li X.-Z.; Wang E.-G.; Jiang Y. Direct visualization of concerted proton tunnelling in a water nanocluster. Nat. Phys. 2015, 11 (3), 235–239. 10.1038/nphys3225.

[ref122] Bozat Ö.; Gedik Z. Spin bath decoherence mediated by phonons. Solid State Commun. 2008, 148 (5), 237–239. 10.1016/j.ssc.2008.08.008.

[ref123] Gomer R. Diffusion of adsorbates on metal surfaces. Rep. Prog. Phys. 1990, 53 (7), 917 10.1088/0034-4885/53/7/002.

[ref124] Lauhon L. J.; Ho W. Direct Observation of the Quantum Tunneling of Single Hydrogen Atoms with a Scanning Tunneling Microscope. Phys. Rev. Lett. 2000, 85 (21), 4566–4569. 10.1103/PhysRevLett.85.4566. 11082597

[ref125] Jewell A. D.; Peng G.; Mattera M. F. G.; Lewis E. A.; Murphy C. J.; Kyriakou G.; Mavrikakis M.; Sykes E. C. H. Quantum Tunneling Enabled Self-Assembly of Hydrogen Atoms on Cu(111). ACS Nano 2012, 6 (11), 10115–10121. 10.1021/nn3038463. 23030641

[ref126] Brumer P.; Shapiro M. Coherence chemistry: controlling chemical reactions [with lasers]. Acc. Chem. Res. 1989, 22, 407–413. 10.1021/ar00168a001.

[ref127] Gordon R. J.; Zhu L.; Seideman T. Coherent Control of Chemical Reactions. Acc. Chem. Res. 1999, 32, 1007–1016. 10.1021/ar970119l.

[ref128] Shabani S.; Darlington T. P.; Gordon C.; Wu W.; Yanev E.; Hone J. C.; Zhu X.; Dreyer C. E.; Schuck P. J.; Pasupathy A. N. Ultralocalized Optoelectronic Properties of Nanobubbles in 2D Semiconductors. Nano Lett. 2022, 22, 7401 10.1021/acs.nanolett.2c02265. 36122409

[ref129] Paul T. Quantum computation and quantum information. Mathematical Structures in Computer Science 2007, 17 (6), 1115–1115. 10.1017/S0960129507006317.

[ref130] Nielsen M. A.; Chuang I. Quantum Computation and Quantum Information. American Journal of Physics 2002, 70 (5), 558–559. 10.1119/1.1463744.

[ref131] Baumgratz T.; Cramer M.; Plenio M. B. Quantifying Coherence. Phys. Rev. Lett. 2014, 113 (14), 140401 10.1103/PhysRevLett.113.140401. 25325620

[ref132] Lombardi F.; Lodi A.; Ma J.; Liu J.; Slota M.; Narita A.; Myers W. K.; Müllen K.; Feng X.; Bogani L. Quantum units from the topological engineering of molecular graphenoids. Science 2019, 366 (6469), 1107–1110. 10.1126/science.aay7203. 31780554

[ref133] Gaita-Ariño A.; Luis F.; Hill S.; Coronado E. Molecular spins for quantum computation. Nat. Chem. 2019, 11 (4), 301–309. 10.1038/s41557-019-0232-y. 30903036

[ref134] Lavroff R. H.; Pennington D. L.; Hua A. S.; Li B. Y.; Williams J. A.; Alexandrova A. N. Recent Innovations in Solid-State and Molecular Qubits for Quantum Information Applications. J. Phys. Chem. B 2021, 125 (44), 12111–12114. 10.1021/acs.jpcb.1c08679. 34758628

[ref135] Hines A. P.; Stamp P. C. E. Decoherence in quantum walks and quantum computers 2008, 86 (4), 541–548. 10.1139/p08-016.

[ref136] von Kugelgen S.; Freedman D. E. A chemical path to quantum information. Science 2019, 366 (6469), 1070–1071. 10.1126/science.aaz4044. 31780541

[ref137] Paul W.; Yang K.; Baumann S.; Romming N.; Choi T.; Lutz C. P.; Heinrich A. J. Control of the millisecond spin lifetime of an electrically probed atom. Nat. Phys. 2017, 13 (4), 403–407. 10.1038/nphys3965.

[ref138] Willke P.; Bilgeri T.; Zhang X.; Wang Y.; Wolf C.; Aubin H.; Heinrich A.; Choi T. Coherent Spin Control of Single Molecules on a Surface. ACS Nano 2021, 15 (11), 17959–17965. 10.1021/acsnano.1c06394. 34767351

[ref139] Regan E. C.; Wang D.; Jin C.; Bakti Utama M. I.; Gao B.; Wei X.; Zhao S.; Zhao W.; Zhang Z.; Yumigeta K.; Blei M.; Carlström J. D.; Watanabe K.; Taniguchi T.; Tongay S.; Crommie M.; Zettl A.; Wang F. Mott and generalized Wigner crystal states in WSe2/WS2 moiré superlattices. Nature 2020, 579 (7799), 359–363. 10.1038/s41586-020-2092-4. 32188951

[ref140] Li H.; Li S.; Regan E. C.; Wang D.; Zhao W.; Kahn S.; Yumigeta K.; Blei M.; Taniguchi T.; Watanabe K.; Tongay S.; Zettl A.; Crommie M. F.; Wang F. Imaging two-dimensional generalized Wigner crystals. Nature 2021, 597 (7878), 650–654. 10.1038/s41586-021-03874-9. 34588665

[ref141] Xu Y.; Liu S.; Rhodes D. A.; Watanabe K.; Taniguchi T.; Hone J.; Elser V.; Mak K. F.; Shan J. Correlated insulating states at fractional fillings of moiré superlattices. Nature 2020, 587 (7833), 214–218. 10.1038/s41586-020-2868-6. 33177668

[ref142] Huang X.; Wang T.; Miao S.; Wang C.; Li Z.; Lian Z.; Taniguchi T.; Watanabe K.; Okamoto S.; Xiao D.; Shi S.-F.; Cui Y.-T. Correlated insulating states at fractional fillings of the WS2/WSe2 moiré lattice. Nat. Phys. 2021, 17 (6), 715–719. 10.1038/s41567-021-01171-w.

[ref143] Li H.; Li S.; Naik M. H.; Xie J.; Li X.; Regan E.; Wang D.; Zhao W.; Yumigeta K.; Blei M.; Taniguchi T.; Watanabe K.; Tongay S.; Zettl A.; Louie S. G.; Crommie M. F.; Wang F. Imaging local discharge cascades for correlated electrons in WS2/WSe2 moiré superlattices. Nat. Phys. 2021, 17 (10), 1114–1119. 10.1038/s41567-021-01324-x.

[ref144] Bi L.; Liang K.; Czap G.; Wang H.; Yang K.; Li S. Recent progress in probing atomic and molecular quantum coherence with scanning tunneling microscopy. Prog. Surf. Sci. 2022, 100696 10.1016/j.progsurf.2022.100696.

[ref145] Kloth P.; Thias T.; Bunjes O.; Haar J. v. d.; Wenderoth M. A versatile implementation of pulsed optical excitation in scanning tunneling microscopy. Rev. Sci. Instrum. 2016, 87 (12), 123702 10.1063/1.4971189. 28040983

[ref146] Rosławska A.; Merino P.; Große C.; Leon C. C.; Gunnarsson O.; Etzkorn M.; Kuhnke K.; Kern K. Single Charge and Exciton Dynamics Probed by Molecular-Scale-Induced Electroluminescence. Nano Lett. 2018, 18 (6), 4001–4007. 10.1021/acs.nanolett.8b01489. 29799760

[ref147] Rosławska A.; Leon C. C.; Grewal A.; Merino P.; Kuhnke K.; Kern K. Atomic-Scale Dynamics Probed by Photon Correlations. ACS Nano 2020, 14 (6), 6366–6375. 10.1021/acsnano.0c03704. 32479059 PMC7315641

[ref148] Mogi H.; Wang Z.-h.; Kikuchi R.; Hyun Yoon C.; Yoshida S.; Takeuchi O.; Shigekawa H. Externally triggerable optical pump-probe scanning tunneling microscopy. Applied Physics Express 2019, 12 (2), 025005 10.7567/1882-0786/aaf8b2.

[ref149] Yoshida S.; Hirori H.; Tachizaki T.; Yoshioka K.; Arashida Y.; Wang Z.-H.; Sanari Y.; Takeuchi O.; Kanemitsu Y.; Shigekawa H. Subcycle Transient Scanning Tunneling Spectroscopy with Visualization of Enhanced Terahertz Near Field. ACS Photonics 2019, 6 (6), 1356–1364. 10.1021/acsphotonics.9b00266.

[ref150] Cocker T.; Jelic V.; Hillenbrand R.; Hegmann F. Nanoscale terahertz scanning probe microscopy. Nat. Photonics 2021, 15 (8), 558–569. 10.1038/s41566-021-00835-6.

[ref151] Martín Sabanés N.; Krecinic F.; Kumagai T.; Schulz F.; Wolf M.; Müller M. Femtosecond Thermal and Nonthermal Hot Electron Tunneling Inside a Photoexcited Tunnel Junction. ACS Nano 2022, 16 (9), 14479–14489. 10.1021/acsnano.2c04846. 36027581 PMC9527804

[ref152] Iwaya K.; Yokota M.; Hanada H.; Mogi H.; Yoshida S.; Takeuchi O.; Miyatake Y.; Shigekawa H. Externally-triggerable optical pump-probe scanning tunneling microscopy with a time resolution of tens-picosecond. Sci. Rep. 2023, 13 (1), 818 10.1038/s41598-023-27383-z. 36697458 PMC9877009

